# Supplementary material for: Sensitivity and specificity of rapid hepatitis C antibody assays in freshly collected whole blood, plasma and serum samples: A multicentre prospective study
Source: PLoS One. 2020 Dec 3;15(12):e0243040. doi: 10.1371/journal.pone.0243040 (PMC7714359; doi:10.1371/journal.pone.0243040)
Supplement: S1 Table — (DOCX) [file pone.0243040.s002.docx]

**Table S1.** Investigational and reference RDT operational characteristics

|  | **HCV-Ab Rapid** | **First Response HCV** | **SD Bioline HCV (reference RDT)** |
| --- | --- | --- | --- |
| Intended users | Professional use | Professional use | Professional use |
| Whole blood sample volume | 50 μl | 35 μl | 10 μl |
| Application method | Provided capillary | Provided pipette | Provided capillary |
| Plasma/serum sample volume | 50 μl | 35 μl | 10 μl |
| Application method | Provided pipette | Provided pipette | Precision pipette |
| Buffer requirements | 1 drop | 1 drop | 4 drops |
| Incubation time | 10 minutes | 20 minutes | 5 minutes |
| Result stability time | 5 minutes | 10 minutes | 15 minutes |
| Plasma/serum 4°C storage stability | 3 days | 3 days | 3 days |
| Kit storage conditions | 2–30°C | 2–30°C | 2–30°C |
| Total test time | 10 minutes | 20 minutes | 10 minutes |
