# Supplementary material for: Sensitivity and specificity of rapid hepatitis C antibody assays in freshly collected whole blood, plasma and serum samples: A multicentre prospective study
Source: PLoS One. 2020 Dec 3;15(12):e0243040. doi: 10.1371/journal.pone.0243040 (PMC7714359; doi:10.1371/journal.pone.0243040)
Supplement: S2 Table — (DOCX) [file pone.0243040.s003.docx]

**Table S2.** HCV genotype of false negative whole blood samples by country

| **Samples per genotype, n** | **Cambodia** | | **Georgia** | | |
| --- | --- | --- | --- | --- | --- |
|  | **HCV-Ab Rapid** | **First Response HCV** | | **HCV-Ab Rapid** | **First Response HCV** |
| 1, 1a, 1b | 7 (1b) | 1 | |  |  |
| 2 | 1 | 1 | | 1 |  |
| 3 |  |  | | 1 | 1 |
| 6 | 2 | 1 | |  |  |
| Mixed |  |  | | 1 | 1 |
| Not determinable | 1 |  | |  |  |

HCV, hepatitis C virus.
