# Supplementary material for: Sensitivity and specificity of rapid hepatitis C antibody assays in freshly collected whole blood, plasma and serum samples: A multicentre prospective study
Source: PLoS One. 2020 Dec 3;15(12):e0243040. doi: 10.1371/journal.pone.0243040 (PMC7714359; doi:10.1371/journal.pone.0243040)
Supplement: S3 Table — (DOCX) [file pone.0243040.s004.docx]

**Table S3.** Performance of the reference RDT test SD Bioline compared with the composite reference standard

|  | **TN, n** | **TP, n** | **FN, n** | **FP, n** | **Sensitivity, % (95% CI)** | **Specificity, % (95% CI)** |
| --- | --- | --- | --- | --- | --- | --- |
| **Overall sample set** | | | | | | |
| Whole blood | 963 | 442 | 47 | 3 | 90.4 (87.5, 92.7) | 99.7 (99.1, 99.9) |
| Plasma | 965 | 465 | 24 | 1 | 95.1 (92.8, 96.7) | 99.9 (99.4, 100) |
| Serum | 964 | 459 | 30 | 2 | 93.9 (91.4, 95.7) | 99.8 (99.2, 99.9) |
| **Samples with detectable VL** | | | | | | |
| Whole blood | — | 299 | 7 | — | 97.7 (95.4, 98.9) | — |
| Plasma | — | 302 | 4 | — | 98.7 (96.7, 99.5) | — |
| Serum | — | 301 | 5 | — | 98.4 (96.2, 99.3) | — |
| **Cambodia** |  |  |  |  |  |  |
| Whole blood | 513 | 177 | 37 | 2 | 82.7 (77.1, 87.2) | 99.6 (98.6, 99.9) |
| Plasma | 514 | 198 | 16 | 1 | 92.5 (88.2, 95.3) | 99.8 (98.9, 100) |
| Serum | 513 | 192 | 22 | 2 | 89.7 (84.9, 93.1) | 99.6 (98.6, 99.9) |
| **Georgia** |  |  |  |  |  |  |
| Whole blood | 450 | 265 | 10 | 1 | 96.4 (93.4, 98.0) | 99.8 (98.8, 100) |
| Plasma | 451 | 267 | 8 | 0 | 97.1 (94.4, 98.5) | 100 (99.2, 100) |
| Serum | 451 | 267 | 8 | 0 | 97.1 (94.4, 98.5) | 100 (99.2, 100) |
| **Performance comparison, p-values** | | | | | | |
| **Sample type (all samples)** | | **Sensitivity** | | | **Specificity** | |
| Whole blood vs plasma | | 0.160 | | | 1.0 | |
| Whole blood vs serum | | 1.0 | | | 1.0 | |
| Plasma vs serum | | 1.0 | | | 1.0 | |
| **Cambodia vs Georgia** | |  | | |  | |
| Whole blood | | <0.001 | | | 1.0 | |
| Plasma | | 0.280 | | | 1.0 | |
| Serum | | 0.012 | | | 1.0 | |

CI, confidence interval; FN, false negative; FP, false positive; TN, true negative; TP, true positive; VL, viral load
