# Supplementary material for: Sensitivity and specificity of rapid hepatitis C antibody assays in freshly collected whole blood, plasma and serum samples: A multicentre prospective study
Source: PLoS One. 2020 Dec 3;15(12):e0243040. doi: 10.1371/journal.pone.0243040 (PMC7714359; doi:10.1371/journal.pone.0243040)
Supplement: S4 Table — (DOCX) [file pone.0243040.s005.docx]

**Table S4.** Multivariable logistic regression analysis for RDT performance in whole blood (p-values)

|  | **HCV-Ab Rapid** | **HCV-Ab Rapid** | **First Response  HCV** | **First Response  HCV** |
| --- | --- | --- | --- | --- |
| **Covariate** | **CRS** | **Reference RDT** | **CRS** | **Reference RDT** |
| Country: Cambodia | 0.002 | 0.009 | 0.006 | 0.027 |
| Gender: male | 0.013 | 0.069 | 0.055 | 0.515 |
| Age | 0.736 | 0.344 | 0.756 | 0.423 |
| Undetectable viral load | 0.275 | 0.997 | 0.990 | 0.998 |
| HCV genotype 1 | 0.416 | 0.997 | 0.992 | 0.998 |
| HCV genotype 2 | 0.663 | 0.997 | 0.991 | 1.000 |
| HCV genotype 3 | 0.562 | 1.000 | 0.991 | 1.000 |
| HCV genotype 6 | 0.140 | 0.997 | 0.992 | 0.998 |
| HCV genotype mixed | 0.972 | 1.000 | 0.990 | 1.000 |

CRS, composite reference standard; HCV, hepatitis C virus
