# Supplementary material for: Sensitivity and specificity of rapid hepatitis C antibody assays in freshly collected whole blood, plasma and serum samples: A multicentre prospective study
Source: PLoS One. 2020 Dec 3;15(12):e0243040. doi: 10.1371/journal.pone.0243040 (PMC7714359; doi:10.1371/journal.pone.0243040)
Supplement: S5 Table — (DOCX) [file pone.0243040.s006.docx]

**Table S5.** Protocol deviations

| **Type of deviation** | **Date** | **Description** | **Action/comment** | **Country** |
| --- | --- | --- | --- | --- |
| Reference testing on frozen samples | 31-07-2019 to  06-10-2019 | In Cambodia, all reference testing was performed on frozen samples for samples collected during this time period due to the pending arrival of test kits. Sample IDs: HC013010001 – 010283 | Samples remained in the study as the reference tests are validated for testing on frozen samples | Cambodia |
| RDT serum/plasma testing | 06-08-2019 to 13-08-2019 | During this period, there were 9 samples for which RDT serum and plasma testing was performed the day after whole blood testing (i.e. not on the same day of whole blood testing) | Samples remained in the study as sample stability is 3 days at 4°C according to the manufacturer’s instructions for use | Cambodia |
| Use of Pasteur pipette for whole blood HCV-Ab Rapid testing | 02-08-2019 to  26-08-2019 | During this period, the site used the kit-provided Pasteur pipette for collection of the whole blood sample, instead of the kit-provided capillary pipette (as per the instructions for use). Sample IDs: HC013020001 - 020080 | There is a risk of inaccurate sample volume. An analysis of FN samples showed that in total this site had 5 FN samples for HCV-Ab Rapid v CRS, 3 of which were among the affected samples cohort. However, 2 of these had an undetectable VL, making it likely that this was the reason for FN. There were no FPs among these samples. All samples remained in the study | Georgia |
| Use of capillary pipette instead of micropipette for reference RDT serum/plasma testing | 02-08-2019 to  26-08-2019 | During this period, the sites used the kit-provided capillary pipette for reference RDT SD Bioline serum/plasma testing instead of a micropipette (as per the instructions for use). Sample IDs: HC013020001 – HC013020080 (CMHPA)  HC013030001 – HC013030161 (NCDC) | There is a risk of inaccurate sample volume. For serum and plasma testing, there were in total 8 FN samples (same IDs for both sample types). Five of 6 samples had an undetectable VL and were also FN for serum/plasma testing with First Response HCV and HCV-Ab Rapid (6/6 for HCV-Ab Rapid plasma), indicating that the FN testing was likely due to low antibody titre. There were no FPs among these samples | Georgia |
| Enrolment criteria | 31-10-2019 | A 16-year old individual was enrolled, which is below the eligible enrolment age (18 years). Sample ID: HC013020287 | The participant was withdrawn from the study and all collected samples destroyed | Georgia |
| Missing reference EIA | 17-12-2019 | For sample ID HC013020450, the reference test Murex EIA was not performed as the sample was forgotten during the last reference test EIA run and the site had run out of EIAs to repeat testing | The sample remained in the study as the Fujirebio EIA and the LIA were both negative and thus the final outcome of the composite reference standard would not have been changed based on the Murex EIA result | Georgia |

EIA, enzyme immunoassay; FN, false negative; FP, false positive; RDT, rapid diagnostic test; VL, viral load
