# Supplementary material for: Sensitivity and specificity of rapid hepatitis C antibody assays in freshly collected whole blood, plasma and serum samples: A multicentre prospective study
Source: PLoS One. 2020 Dec 3;15(12):e0243040. doi: 10.1371/journal.pone.0243040 (PMC7714359; doi:10.1371/journal.pone.0243040)
Supplement: S1 File — (PDF) [file pone.0243040.s007.pdf]

CLINICAL STUDY PROTOCOL

Protocol Title

Prospective diagnostic accuracy study of rapid diagnostic tests (RDTs) detecting antibodies against hepatitis C virus (HCV) in freshly collected whole blood, plasma and serum

Short title

Evaluation study of HCV RDTs in fresh samples

Protocol Version Number:

8162-2/2 version 1.0

Date:

26-Mar-2019

Disease Programme:

HCV

FIND
Campus Biotech
Chemin des Mines 9
1202 Geneva, Switzerland
T: +41 (0)22 710 05 90

CONFIDENTIAL

Confidentiality Statement:

The information contained in this document, especially unpublished data, is the property of FIND (or under its control) and may not be reproduced, published or disclosed to others without prior written authorization from FIND.

Table of Contents

Institutions/Organizations/Partners involved in the study **5**
Signatures (Sponsor) **6**
Statement of Principal Investigator **7**
Protocol history **8**
Abbreviations **9**
Protocol synopsis **10**
Schedule of assessment **13**
1 Introduction **14**
1.1 Study rationale 14
1.2 Background 15
1.3 Risk/benefit assessment 15
2 Study objectives and endpoints **16**
3 Study design **17**
3.1 General design 17
3.2 Scientific rationale for study design 19
3.3 End of study definition 20
3.4 Study sites, population and eligibility 20
4 Study intervention **21**
4.1 Investigational product (or Study intervention) 22
4.2 Preparation/Handling/Storage/Accountability 23
4.3 Minimisation of error and bias 24
5 Participant discontinuation/withdrawal **25**
5.1 Participant discontinuation/Withdrawal from the study 25
5.2 Loss to follow up 25
6 Study procedures **25**
6.1 Participant enrolment 27
6.2 RDT testing 27
6.3 Venous blood draw and aliquot preparation 27
6.4 Reference and confirmatory testing at reference laboratories 28
6.5 Specimen collection, handling, transport and storage 28
6.6 Index test and reference standard test procedures 29
6.7 Confirmatory testing and genotyping 30
6.8 Experimental Biomarkers 30
6.9 Other tests 31

6.10 Safety assessments ... 31
6.11 Other study procedures ... 31
7 Safety and incident reporting ... 31
7.1 Medical device incidents (including malfunctions) ... 31
8 Statistical considerations ... 33
8.1 Statistical hypotheses ... 33
8.2 Sample size determination ... 33
8.3 Populations for analyses ... 34
8.4 Statistical analysis plan ... 34
8.5 Planned interim analyses ... 36
9 Regulatory and ethical considerations ... 37
9.1 Regulatory and ethics approvals ... 37
9.2 Financial disclosure ... 37
9.3 Informed consent process ... 38
9.4 Data protection ... 38
10 Data handling and record keeping ... 39
10.1 Source data and source documents ... 39
10.2 Data management ... 40
11 Quality management ... 42
11.1 Quality control (monitoring) ... 42
11.2 Quality assurance (auditing) ... 42
11.3 Study and site closure ... 42
12 Publication policy ... 43
13 References ... 44
14 Appendices ... 46
Appendix 1: Safety definitions and reporting ... 46
Appendix 2: Incident definition and reporting ... 47
Appendix 3: Technical appraisal form ... 48
Appendix 4: Summary of changes ... 49

Institutions/Organizations/Partners involved in the study

**Organization/Institution/Company/Partner***	**Role in the Study**
Foundation for Innovative New Diagnostics (FIND), Geneva, Switzerland	Sponsor
National Center for Disease Control & Public Health (NCDC), Lugar Centre, Tbilisi Georgia (reference laboratory)<br>Opioid Substitution Treatment Centre (primary healthcare facility)<br>NCDC Screening Centre (primary healthcare facility)	Investigational sites
Sihanouk Hospital Center of Hope (SHCH), Phnom Penh, Cambodia (reference laboratory)<br>SHCH Outpatient Clinic (primary healthcare facility)	Investigational site
Beijing Wantai Biological Pharmacy Enterprise, China	IVD manufacturer
Premiere Medical Corporation, India	IVD manufacturer
AccessBio, USA	IVD manufacturer
ZeptoMetrix, USA	Biorepository

FIND (Sponsor)

Contact person: Dr. Beatrice Vetter

Campus Biotech, Chemin des Mines 9

1202 Geneva, Switzerland

National Center for Disease Control & Public Health (NCDC): Lugar Center (reference laboratory)

Principle Investigator: Dr. Maia Alkhazashvili

99 Kakheti Highway

Tbilisi 0198 Georgia

- **Opioid Substitution Treatment Centre** (primary healthcare facility)
  Center for Mental Health and Prevention of Addiction LTD
  21a, Kavtaradze Street, Tbilisi, Georgia, represented by its Director Lasha Kiladze
- **NCDC Screening Centre** (primary healthcare facility)
  9, Asatiani Street, Tbilisi, Georgia

Sihanouk Hospital Center of Hope (SHCH) (reference laboratory)
Principle Investigator: Dr. Sokkab An

Department of Infectious Diseases

St. 134, Sangkat Vealvong, Khan 7 Makara

Phnom Penh, Cambodia

Sub-Investigator: Dr. Anja De Weggheleire
Institute of Tropical Medicine HIV/STD Reference Laboratory Kronenburgstraat 43, 2000 Antwerp Belgium

- **SHCH Outpatient Clinic** (primary healthcare facility)

---

*Terms of references and nature of agreements are available from FIND upon request.

Signatures (Sponsor)

We, the undersigned, have developed, reviewed and approved this protocol, including appendices. We will supervise and coordinate the clinical study according to the principles outlined in the Declaration of Helsinki and Good Clinical Practice and in compliance with applicable regulatory requirements.

SENOR SCIENTIFIC OFFICER HCV PROGRAMME

Name:

Signature: ______________________________ Date: ____________________

DD/MMM/YYYY

HEAD OF CLINICAL & REGULATORY AFFAIRS

Name:

Signature: ______________________________ Date: ____________________

DD/MMM/YYYY

STUDY MANAGER

Name:

Signature: ______________________________ Date: ____________________

DD/MMM/YYYY

HEAD OF DATA SERVICES & BIOBANKING

Name:

Signature: ______________________________ Date: ____________________

DD/MMM/YYYY

Statement of Principal Investigator

In signing this page, I, the undersigned, agree to conduct the study according to the protocol and ICH-GCP E6 (R2) guidelines and in compliance with applicable regulations.

I will ensure that the requirements relating to obtaining Institutional Review Board (IRB)/ Independent Ethics Committee (IEC) review and approval are met. I will promptly report to the IRB/IEC any and all changes in the research activities covered by this protocol.

I have sufficient time to properly conduct and complete the study within the agreed study period and I have adequate resources (staff and facilities) for the foreseen duration of the study.

I am responsible for supervising any individual or party to whom I delegate study related duties and functions conducted at the study site. Further, I will ensure this individual or party is qualified to perform those study-related duties and functions.

I certify that key individuals involved with the conduct of this study, including myself, have completed GCP training and, if applicable, Human Subjects Protection Training.

I understand that all information obtained during the conduct of the study with regard to the subjects' state of health will be regarded as confidential. No participant's names or personal identifying information may be disclosed. All participant data will be anonymized and identified by assigned numbers on all Case Report Forms, laboratory samples and other study related information (such as essential documents) forwarded to FIND. Monitoring and auditing by FIND, and inspection by the appropriate regulatory authority(ies), will be permitted.

I will maintain confidentiality of this protocol and all other related investigational materials. Information taken from the study protocol may not be disseminated or discussed with a third party without the express consent of FIND.

Name of Principal Investigator: ____________________________________
(Print)

Signature: ______________________________________ Date: ________________________

DD/MMM/YYYY

Protocol history

Version number	*Release date*	*Comments*
1.0	26-Mar-2019	Initial version

See Appendix 4, Summary of changes

Abbreviations

Abbreviation/acronym	*Meaning*
AE	adverse event
CRF	case report form
EDC	electronic data capturing
FIND	Foundation for Innovative New Diagnostics
GCP	Good Clinical Practice
GCLP	Good Clinical Laboratory Practice
HCC	hepatocellular carcinoma
HCV	hepatitis C virus
HIC	high income country
HIV	human immunodeficiency virus
ICF	Informed Consent Form
IFU	instructions for use
IEC	Independent Ethics Committee
ICH	International Council on Harmonisation
IRB	Institutional Review Board
ISO	International Organization for Standardization
LMIC	low- and middle-income country
NCDC	National Center for Disease Control & Public Health
NPV	negative predictive value
OST	opioid substitution treatment
PPV	positive predictive value
PQ	pre-qualification
QA	quality assurance
QC	quality control
QMS	quality management system
RA	regulatory authority
RDT	rapid diagnostic test
REF	result entry form
RLS	resource-limited setting
RM	risk management
RRF	result report form
SAP	statistical analysis plan
SAE	serious adverse event
sens/spec	sensitivity/specificity
SOP	standard operating procedure
SHCH	Sihanouk Hospital Center of Hope
TMF	trial master file
+ve	positive
-ve	negative
WHO	World Health Organization

Protocol synopsis

Title	Prospective diagnostic accuracy study of rapid diagnostic tests (RDTs) detecting antibodies against hepatitis C virus (HCV) in freshly collected whole blood, plasma and serum
Short title	Evaluation study of HCV RDTs in fresh samples
Version and date	8162-2/2 version 1.0; 26-Mar-2019
Background and rationale	Hepatitis C virus (HCV) infection is a major public health burden, with an estimated 71 million patients being infected globally. If undiagnosed, HCV infection can lead to severe liver damage, including hepatocellular carcinoma (HCC). The World Health Organization (WHO) recently set a target to eliminate HCV by 2030. The first critical step in reaching this target is to accurately identify people infected with HCV. Globally, significant gaps remain in diagnosis of HCV, with four out of five people infected still unaware of their status, largely due to lack of access to testing services.<br>In resource-limited settings (RLS), laboratory-based testing remains a mainstay for HCV screening and confirmatory testing. Such laboratory-based assays rely on transportation of temperature-sensitive samples from clinic sites to centralized laboratories, high-tech equipment and highly skilled laboratory technicians, limiting the scope of access to testing and resulting in long turn-around times for results. In recent years, new technologies have been developed to decentralize HCV screening and confirmatory testing using point-of-care (POC) assays to overcome these barriers and improve patient outcomes.<br>Rapid diagnostic tests (RDT) for screening for HCV are affordable, accurate, easy to use by healthcare workers, and robust in field settings. Although a number of HCV RDTs are on the market, currently only two have received WHO pre-qualification (PQ) status, demonstrating their accuracy and reliability for use in the field in RLS. Data on a number of other RDTs indicate their suitability for use. More data is needed, however, in field settings for these HCV RDTs, to demonstrate their accuracy and quality in end-user studies and to provide evidence for WHO PQ approval.<br>Based on the results of a recent FIND laboratory evaluation assessing the performance of 13 HCV RDTs using archived plasma samples, we have selected three candidate HCV RDTs (“study RDTs”) for further evaluation in field settings (e.g. primary healthcare facilities). The performance of these RDTs measured in frozen plasma specimens indicated that they have a high potential to meet WHO PQ performance criteria in freshly collected samples. In addition, the manufacturers each demonstrate evidence of engagement to pursue both CE-mark status and the WHO PQ process, showing commitment to delivering quality-assured tests to the market.<br>The data generated during this study will be used to inform national and international stakeholders on HCV RDT performance in field settings and shared with manufacturers to support the evidence package for WHO PQ and/or CE-marking.

Primary objective(s)	1.1 To determine the performance of each study RDT in fingerstick whole blood, EDTA plasma and serum in field settings, using a combination of two enzyme immunoassays (EIAs) and a line immunoassay (LIA) as composite reference standard.
Secondary objective(s)	2.1 To determine the performance of each study RDT in fingerstick whole blood, EDTA plasma and serum in field settings, using an RDT already pre-qualified by WHO (“WHO PQed RDT”) as reference standard.<br>2.2 To evaluate the operational characteristics of study RDTs.
Primary endpoints (outcomes)	1.1 Point estimates of sensitivity, specificity, positive and negative predicative values (with 95% confidence intervals) and Cohen’s Kappa Coefficient (κ) of inter-rater agreement for each RDT, using a combination of two EIAs and an LIA as a composite reference standard for the detection of anti-HCV antibodies in fingerstick whole blood, EDTA plasma and serum.
Secondary endpoints (outcomes)	2.1 Point estimates of sensitivity, specificity, positive and negative predicative values (with 95% confidence intervals) and Cohen’s Kappa Coefficient (κ) of inter-rater agreement for each RDT, using the WHO-PQ RDT as reference standard for the detection of anti-HCV antibodies in fingerstick whole blood, EDTA plasma and serum.<br>2.2 Operational characteristics and usability of study RDTs:<br>- Rate of invalid test results /errors: Percentage of invalid test results/errors by RDT, site and error type.<br>- Technical appraisal rating on kit instructions, labelling and test conduct, on a Likert scale.
Study design	This is a prospective multi-centre diagnostic accuracy study. The performance of the study RDTs will be assessed on fingerstick whole blood, EDTA plasma and serum in primary healthcare facilities (field settings) compared to a composite laboratory reference standard and an RDT that is WHO pre-qualified (Abbott SD Bioline HCV Test).<br>The study is aligned with WHO PQ performance evaluation criteria as per the WHO Technical Specification Series document TSS-7: “Rapid diagnostic tests to detect hepatitis C antibody or antigen” (still in draft).
Study sites/setting	Primary healthcare facilities and associated reference laboratories in areas of high and low HCV prevalence<br>• Country 1: Georgia<br>- Opioid Substitution Treatment Centre (primary healthcare facility)<br>- National Centre for Disease Control (NCDC) screening centre (primary healthcare facility)<br>- NCDC Lugar Centre (reference laboratory)<br>• Country 2: Cambodia<br>- Sihanouk Hospital Centre of Hope (SHCH) outpatient clinic (primary healthcare facility)<br>- SHCH reference laboratory

Study population	Individuals attending the above primary healthcare facilities (related or un-related to HCV) and with known or unknown HCV status.
Sample Size	In the previous FIND HCV RDT study, the average performance of sensitivity and specificity in EDTA plasma of the three selected tests was 96.3% and 98.3%, respectively. Based on this, a conservative assumption on performance in fresh samples in the hands of the intended user was made, setting the expected average sensitivity and specificity to 90% in whole blood and 95% in EDTA plasma and serum.<br>To obtain a reliable performance estimate, the following sample sizes were determined:<br><u>Sensitivity:</u><br>• 440 whole blood HCV antibody positive<br>• 440 EDTA plasma HCV antibody positive<br>• 440 serum HCV antibody positive<br>note: a total of 440 participants will be enrolled and each participant will provide whole blood, EDTA plasma and serum samples<br><u>Specificity:</u><br>• 1’100 whole blood HCV antibody and HCV RNA negative<br>• 1’100 EDTA plasma HCV antibody and HCV RNA negative<br>• 1’100 serum HCV antibody and HCV RNA negative<br>Note: a total of 1’100 participants will be enrolled and each participant will provide whole blood, EDTA plasma and serum samples<br>These sample sizes meet the requirements of the WHO Technical Specification Series document TSS-7: “Rapid diagnostic tests to detect hepatitis C antibody or antigen” (still in draft) for evaluation of clinical sensitivity and specificity.<br>Each primary healthcare facility will have set enrolment targets to avoid unbalanced recruitment.
Eligibility criteria	Age: ≥ 18 years<br>No history of HCV treatment<br>Willing to participate and able to give consent<br>Willing to get tested for HIV
Study duration	9 months
Time schedule	Training month 1<br>Recruitment and testing months 2-7<br>Statistical analysis months 8-9

Schedule of assessment

***Table 1**: Schedule of assessment*

	Daily activities per participant in primary healthcare sites		Weekly activities in reference laboratories	
**Recruitment**	Day 1	Assessment of eligibility		
	Day 1	Obtaining informed consent		
	Day 1	Collection of demographic information		
**Testing procedures** *with* **participant present**	Day 1	Fingerstick whole blood testing on three study HCV RDTs and one WHO PQed RDT		
	Day 1	Fingerstick whole blood testing on HIV RDT		
	Day 1	Collection of venepuncture blood in EDTA plasma and serum tubes		
**Testing procedures** *without* **participant present**	Day 1	Centrifugation and separation of EDTA plasma and serum	Days 2-7	HCV and HIV RNA confirmatory testing of RDT-positive samples
	Day 1	Preparation of EDTA plasma and serum aliquots	Days 2-7	EDTA plasma EIA/LIA reference testing
	Day 1	EDTA plasma and serum testing on three study HCV RDTs and one WHO-PQ RDT		
**Data entry**	Day 1-7	Transcription of Participant Worksheet information and results into Open Clinica		

1 Introduction

The World Health Organization (WHO) guidelines for testing of hepatitis C virus (HCV) infection recommend the use of a single quality-assured serological in vitro diagnostic (IVD) test, either a laboratory-based immunoassay or rapid diagnostic test (RDT) to detect HCV antibodies [1]. A point-of-care (POC) RDT should fulfil the ASSURED criteria, defined by the WHO as being affordable, sensitive, specific, user-friendly, rapid and robust, equipment-free and deliverable to the end-user [2]. RDT results can be provided in under 30 minutes, which allows testing, counselling, and referral of the patient in one visit.

In resource-limited settings (RLS), RDTs present an attractive alternative to laboratory-based immunoassays, due to their affordability, ease of use and ability for testing on various samples, including plasma, serum, fingerstick whole blood or oral fluid. For any diagnostic test, whether performed in a laboratory or at the point of care, quality assurance is of major importance to obtain an accurate and reliable RDT result. However, of the many available RDT products for detecting HCV antibodies, only two currently have received WHO pre-qualification (PQ) status. WHO PQ status is a means to ensure appropriate performance in populations of low and middle income countries (LMICs) in the most relevant samples and settings, e.g. fresh fingerstick whole blood in primary healthcare facilities or other field settings [3].

1.1 Study rationale

FIND has previously conducted an extensive evaluation study of 13 HCV RDTs in reference laboratories on archived EDTA plasma samples from LMICs, with the aim to identify well-performing test that could meet WHO PQ requirements (FIND "Evaluation study of HCV RDTs", no. 8162-2/1 [4]). A total of 1'800 archived EDTA plasma samples were analysed (800 HCV antibody +ve, 1'000 HCV antibody-ve), originating largely from LMICs (Nigeria, Georgia, Cambodia) and to a small percentage from a high income country (HIC; Belgium 6%). Half of all samples were from HIV co-infected patients (HCV+ve and HCV-ve) to determine performance in samples from patients with this frequently found co-infection. Preliminary results of this study showed that all RDTs met WHO PQ performance requirements in samples from patients not co-infected with HIV (98% sensitivity and 97% specificity [5]). In samples from HIV co-infected patients, however, performance was highly variable, with several tests showing over 10% reduced sensitivity for HCV antibodies in the presence of HIV infection (preliminary, unpublished results).

Three of the 13 RDTs from this initial study had a comparatively high performance in the HCV-mono and HCV/HIV co-infected populations, leading to an acceptable overall sensitivity and specificity for further evaluation: Beijing Wantai HCV-Ab Rapid Test ("Wantai") (sens/spec 97.1%/97.5%); Premier Medical Corporation First Response HCV Card Test ("PMC") (sens/spec 95.9%/99.5%); Access Bio CareStart HCV Antibody Test ("AccessBio") (sens/spec 96.1%/97.8%).

The current study aims to conduct a clinical evaluation of these three tests on fresh whole blood, EDTA plasma and serum samples in primary healthcare settings to evaluate performance as per WHO PQ requirements [6]. The Abbott SD Bioline HCV test will be used as the comparator in the

primary healthcare setting, to compare the performance of the study RDTs to an RDT that is pre-qualified by the WHO and to enable direct comparison of RDT performance in whole blood.

The manufacturers of the three study RDTs wish to seek WHO PQ approval, have easy-to-perform tests (two steps) and are offering their current HCV RDT at low prices, making them good candidates for procurement in LMICs. The data generated during this study can be used to seek WHO pre-qualification and/or CE-marking.

1.2 Background

In 2015, an estimated 71 million people had chronic hepatitis C infection worldwide and viral hepatitis caused 1.34 million death [7]. The recent introduction of direct-acting antivirals (DAAs) has led to cure in more than 90% of treated individuals [8, 9] and in 2016, the WHO released updated guidelines for the screening and treatment of people with chronic hepatitis C infection [10].

Georgia has one of the highest HCV prevalence globally, with an estimated 5.4% of the adult population chronically infected and a national seroprevalence of 7.7% [11]. The most predominant HCV genotypes are genotype 1b, 2, 3 and recombinants of 1b/2 [4, 11]. The HCV prevalence in Cambodia is estimated to be 2.3%, with the most predominant genotypes being genotype 1b and 6 [12, 13]. Genotypes 1a can also be found in both countries [4].

The lack of quality-assured RDTs for serology testing is an important barrier to large scale access to HCV diagnostics and as such to most effective HCV programme implementation in high burden countries [14]. Some RDTs show high accuracy compared to laboratory-based methods [15, 16] but many RDTs used in LMICs are without stringent regulatory approval, therefore their quality often remains unknown.

1.3 Risk/benefit assessment

Risks associated with participation in this study are minimal and are restricted to the sample collection procedure: The fingerstick blood samples will be collected by pricking one or two fingers at least once, or as required, to obtain enough blood to perform all RDTs. An additional blood sample will be taken by a single venepuncture to allow for confirmatory and reference testing. Physical discomfort, transient bleeding and bruising may result when blood is obtained by venepuncture. Aseptic techniques and universal precautions against body fluid exposures will be practiced in obtaining blood samples

All enrolled participants with a reactive HCV and/or HIV serology screening result will receive standard of care confirmatory testing.

Participants who attend the primary healthcare facility with the intention to be tested for HCV may benefit from the additional HCV RDTs performed, in case the standard of care HCV RDT has a lower sensitivity, compared to the study RDTs.

Participants at the Sihanouk Hospital Centre of Hope in Cambodia will benefit from free-of-charge HCV and HIV screening and confirmatory testing, for which they normally have to provide a co-pay. Furthermore, all participants with confirmed HCV infection (detectable HCV VL) will receive free-of-charge HCV treatment (HIV treatment is provided at no cost at the hospital).

In Georgia, all HCV and HIV screening, confirmation and treatment is free of charge.

There is a benefit of the study to society as a whole through publication of study results and the use of the data by the manufacturer to seek WHO PQ approval and/or CE marking. The availability of more quality-assured HCV RDTs will enable better screening, and support identification of undiagnosed HCV infected individuals.

2 Study objectives and endpoints

***Table 2**: Study objective and endpoints*

Objectives	*Endpoints*
Primary	
1.1 To determine the performance of each study RDT in fingerstick whole blood, EDTA plasma and serum in field settings, using a combination of two enzyme immunoassays (EIAs) and a line immunoassay (LIA) as composite reference standard.	1.1 Point estimates of sensitivity, specificity, positive and negative predicative values (with 95% confidence intervals) and Cohen's Kappa Coefficient (κ) of inter-rater agreement for each RDT, using a combination of two EIAs and a LIA as composite reference standard for the detection of anti-HCV antibodies in fingerstick whole blood, EDTA plasma and serum.
Secondary	
2.1 To determine the performance of each study RDT in fingerstick whole blood, EDTA plasma and serum in field settings, using an RDT already pre-qualified by WHO ("WHO PQed RDT") as reference standard.<br>2.2 To evaluate the operational characteristics of study RDTs.	2.1 Point estimates of sensitivity, specificity, positive and negative predicative values (with 95% confidence intervals) and Cohen's Kappa Coefficient (κ) of inter-rater agreement for each RDT, using the WHO-PQed RDT as reference standard for the detection of anti-HCV antibodies in fingerstick whole blood, EDTA plasma and serum.<br>2.2 Operational characteristics and usability of study RDTs:<br>- Rate of invalid test results /errors: Percentage of invalid test results/errors by RDT, site and error type<br>- Technical appraisal rating on kit instructions, labelling and test conduct, on a Likert scale

3 Study design

3.1 General design

This is a prospective multicentre diagnostic accuracy study. The study will be conducted at primary healthcare facilities for participant recruitment and RDT testing, and at diagnostic reference laboratories for confirmatory and reference testing (see ***Error! Reference source not found.***).

***Figure 1**: Overview of the general study design*

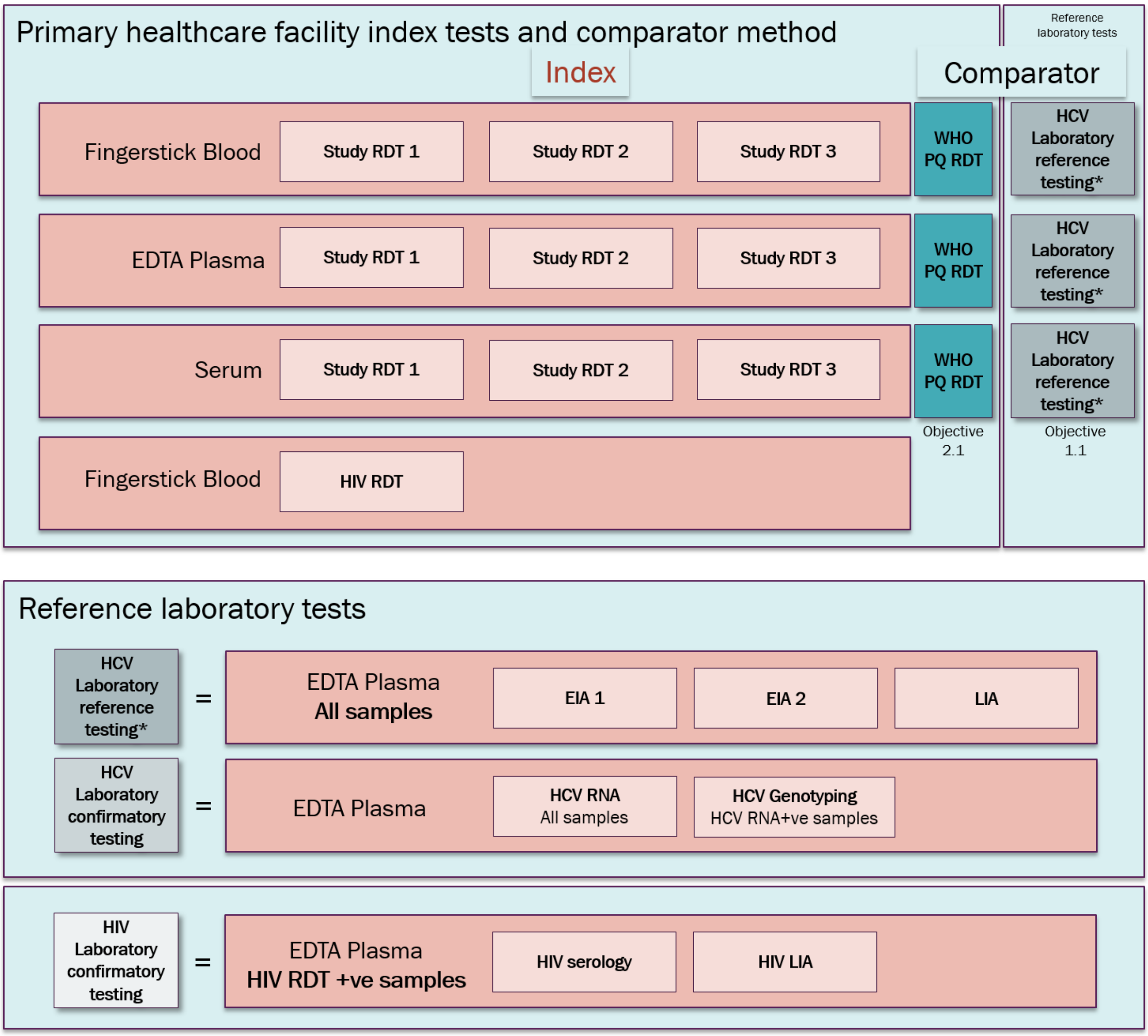

* HCV Reference testing: Enzyme Immunoassays (EIA) Diasorin Murex Anti-HCV v4.0 and Fujirebio INNOTEST HCV Ab IV; Line Immunoassay (LIA): Fujirebio INNO-LIA HCV

In Georgia, the study will be conducted at two primary healthcare facilities. One site offers opioid substitution therapy (OST) and is seeing a population with high HCV prevalence (approximately 40%). The other site is a hepatitis/HIV screening facility for the general population with a lower HCV prevalence of approximately 5%. The latter facility was chosen for the necessary number of

negative samples to be collected. The reference laboratory is the Lugar Centre, which acts as the confirmatory laboratory for both sites under the standard of care.

In Cambodia the study will be conducted at the Sihanouk Hospital Centre of Hope where the general population attends for HIV/HCV screening as well as other medical issues. The reference laboratory is the hospital's diagnostic routine laboratory. HCV viral load and HCV genotyping will be outsourced to the Institut Pasteur du Cambodge in Phnom Penh via service contract, as the Sihanouk Hospital Centre of Hope has no capacities to perform these tests (collectively referred to as "reference laboratory" in this protocol).

Participants will be enrolled sequentially as they present at the primary healthcare facilities. Any individual attending the healthcare facilities will be given the opportunity to participate in the study, regardless of the reason for the visit. At the OST site in Georgia and the outpatient clinic in Cambodia, patients with known HCV-positive serology status will be contacted and invited to participate in the study. Participants will be pre-screened for eligibility and eligible participants who wish to participate in the study will undergo the consent procedure (see section 9.3. Informed consent process) prior to the collection of demographic and clinical information, and samples for study.

3.1.1 RDT testing

For all participants, fingerstick whole blood, EDTA plasma and serum will be tested on the RDTs under evaluation (Wantai, PMC, AccessBio) and on the WHO PQed RDT (SD Bioline) at the primary healthcare facilities. All tests are design-locked, on-market products.

Furthermore, all participants with currently unknown HIV status will be tested for HIV using the locally available HIV RDT. Currently unknown HIV status is defined as not having a medically recorded HIV diagnosis in the healthcare facilities patient registry or having had a negative HIV test more than 12 months ago.

Invalid RDT results will be repeated immediately.

The results will be read and recorded by an operator into a Participant Worksheet and subsequently entered into the electronic case report form (eCRF) in Open Clinica, an electronic data capturing (EDC) system.

3.1.2 Study-specific reference testing (composite reference standard):

All EDTA plasma samples will be subject to study-specific parallel reference testing with two enzyme immunoassays (EIAs) – Diasorin Murex Anti-HCV v4.0 and Fujirebio INNOTEST HCV Ab IV, and one line immunoassay (LIA) – Fujirebio INNO-LIA HCV Score. The aim is to verify HCV antibody status in the samples and characterise the positive samples for antigen-reactivity. All three tests are CE-marked and both EIAs are pre-qualified by the WHO. This reference test combination was also used in the previous FIND HCV RDT study [4].

Reference testing will be performed on fresh (non-frozen) samples in batches within the allowed 4°C storage time as per the manufacturer's instructions for use (IFU) (7 days). EIA test results will be entered into study-specific electronic result entry forms (REF) prior to upload into Open Clinica (by FIND). LIA test results will be entered into paper result report forms (RRFs) and subsequently transcribed into electronic REFs prior to upload into Open Clinica (see section 10.2 on Data management).

3.1.3 Standard of care confirmatory testing

Any initially reactive HCV RDT results on fingerstick whole blood, EDTA plasma or serum, regardless if obtained by a study RDT or the WHO PQed RDT, will be subject to HCV PCR confirmatory testing.

For Georgia, this is done at the reference laboratory (Lugar Centre), using the Abbott RealTime HCV assay for viral load (on EDTA plasma). For Cambodia, this is done at the Institut Pasteur du Cambodge (ISO 15189 accredited, in Phnom Penh) via service contract. Furthermore, the respective laboratories will perform HCV genotyping on all samples with detectable HCV viral load. All results will be entered into the study-specific eCRF and the patient file.

Initially reactive HIV tests will also be subject to local standard of care confirmatory testing.

All patients who are confirmed HCV or HIV positive, will be linked to local healthcare services.

3.2 Scientific rationale for study design

Both study sites are located in resource-limited settings, which is the intended use setting for HCV RDTs to be pre-qualified by the WHO [3]. RDT testing will be performed by intended users of the tests, i.e. primary healthcare workers who routinely perform blood sampling procedures on patients, but are not experienced in performing sophisticated diagnostic laboratory testing.

Matched fingerstick whole blood, EDTA plasma and serum sample will be assessed to evaluate the performance in all sample types claimed by the RDT manufacturers. Performance of RDTs in these sample types will be compared to a composite reference standard as well as to an HCV RDT already pre-qualified by WHO (SD Bioline).

The use of a composite reference standard designed of two EIAs and one LIA, allows accurate characterisation of HCV antibody status (see section 6. Study Procedure for further detail). This method is also used by WHO PQ for sample characterisation during independent laboratory evaluations of RDTs [17]. Using a WHO-PQed HCV RDT (SD Bioline) will allow direct performance comparison in all sample types in the intended use setting.

Assessment of HCV viral load will provide additional information on the status of the infection of the patient, i.e. active or resolved. This information will be used to support the interpretation of discordant results, as some RDTs can have lower sensitivity in patients with resolved HCV infection [18].

Genotyping of HCV positive samples will allow to assess RDT performance in relation to different HCV genotypes. Study sites were chosen to ensure genotype diversity; the most prevalent genotypes in Georgia and Cambodia are 1b, 2, 3 and 1b, 6, respectively [11-13].

Testing study participants for HIV will provide important additional information, as some RDTs have shown lower performance in patients co-infected with HIV during the previous FIND HCV RDT study [4]. Furthermore, it is known that immunocompromised patients may have lower HCV antibody titres [19, 20], potentially impacting RDT performance.

3.3 End of study definition

A participant is considered to have completed the study if he/she has completed the last scheduled procedure shown in the schedule of assessment (Table 1). The end of the study is defined as the date when the last sample was analysed with the composite reference standard tests and all data have been entered into the defined electronic format by the study site (see also section 10.2 Data management).

3.4 Study sites, population and eligibility

3.4.1 Study sites and population

<u>Georgia:</u>

The OST facility in Georgia sees approximately 30 individuals per day with an HCV prevalence of around 40%. Individuals visiting the OST facility are former drug users seeking opioid substitution or general healthcare services. All individuals visiting the facility will be offered to participate in the study, until enrolment targets for HCV negative participants are reached (see Table 5 for enrolment targets). Once these are reached, only individuals with known positive HCV status will be enrolled in the study. From the beginning of enrolment, known HCV positive individuals registered in the OST facility database, will be contacted and invited to participate in the study (see also section 4.3.2 Potential bias). Travel of these patients will be reimbursed according to local inner-city public transport fares.

The NCDC screening facility is near the OST site (approximately 1 km distance) and sees around 20 individuals per day with a 5% prevalence for HCV. Individuals attending the NCDC screening facility are from the general population and wish to have their HCV, HIV or HBV status checked. This screening facility was set up as part of the Georgia HCV elimination programme, which includes awareness raising of free-of-charge screening services. Both screening sites provide the same level of service and are equally competent in terms of HCV/HIV/HBV screening and venous blood draw for confirmatory testing.

Samples are transported daily to the NCDC Lugar Centre reference laboratory by a dedicated sample transport service. The distance from the healthcare facility to the sites is approximately 10 km.

<u>Cambodia:</u>

Sihanouk Hospital Center of Hope is a non-governmental hospital located in Phnom Penh providing low cost medical care for the poor and disadvantaged. They have a general outpatient primary healthcare clinic, which will be the recruitment site for this study. Per day, approximately 175 patients visit the outpatient clinic and the estimated HCV prevalence is 5%.

All individuals visiting the outpatient clinic will be offered to participate in the study, until enrolment targets for HCV negative participants are reached (see Table 5 for enrolment targets). Once these targets are reached, only individuals with known positive HCV status will be enrolled in the study. From the beginning of enrolment, known HCV positive individuals registered in the outpatient clinic database, will be contacted and invited to participate in the study (see also section 4.3.2 Potential bias). Travel of these patients will be reimbursed according to local inner-city public transport fares.

The outpatient clinic is located in close proximity to the reference laboratory, allowing daily transport of samples from the clinic to the laboratory.

3.4.2 Eligibility criteria

Individuals are eligible to participate in the study if all of the following criteria apply:

- ≥ 18 years of age
- Known or unknown HCV serology
- No history of past or present HCV treatment
- Willing to undergo the information and consenting procedure and subsequently have enough time to participate in the study
- Willing to provide 13 ml venepuncture blood sample and a minimum of four whole blood fingerstick samples
- Willing to perform an HIV test
- Individuals can already be registered at the local site or register for the first time when enrolling in the study

Note: Prospective approval of protocol deviations to recruitment and enrolment criteria, also known as protocol waivers or exemptions, is not permitted.

3.4.3 Exclusion criteria

Participants are excluded from the study if any of the following exclusion criteria apply:

- Not able to consent themselves

3.4.4 Screen failures

Screen failures are defined as participants who consent to participate in the clinical study but are not subsequently undergoing any or all study procedures. Participants who do not meet the criteria for participation in the study (screen failure) may be rescreened.

A screen failure for this study would be a participant who agreed to provide a venepuncture blood sample but from whom it was subsequently not possible to obtain this sample due to difficult venous access or needle phobia.

Furthermore, a participant is classified as screen failure if information provided is subsequently discovered to be incorrect (e.g. false medical history).

4 Study intervention

Study intervention is defined as any investigational intervention(s), marketed product(s), or medical device(s) intended to be used with a study participant according to the study protocol.

This diagnostic accuracy study aims to evaluate performance of HCV RDTs independently of the routinely used HCV RDTs at the study sites. Test results of the study RDTs or final results of this study will not influence standard medical care provided at the site.

4.1 Investigational product (or Study intervention)

The investigational products (index tests) used in this study and key operational characteristics are listed in Table 3. All tests detect antibodies to HCV antigens.

***Table 3**: Investigational products and key operational characteristics*

	Wantai	PMC	AccessBio
**Sample types**	Whole blood, serum, EDTA plasma	Whole blood, serum, EDTA plasma	Whole blood, serum, EDTA plasma
**Intended users**	Professional use	Professional use	Professional use
**Whole blood sample volume**	One drop	One drop	One drop
Application method	Provided pipette	Provided pipette	Provided pipette
**Plasma/serum sample volume**	50 µl	35 µl	10 µl
Application method	Provided pipette	Provided pipette	Provided pipette
**Buffer requirements**	1 drop	1 drop	2 drops
**Incubation time**	10 min	20 min	15 min
**Result stability time**	5 min	10 min	10 min
**Plasma/serum 4°C storage stability**	3 days	3 days	3 days
**Kit storage conditions**	2-30°C	2-30°C	2-30°C
**Total test time**	15 min (negatives need re-checking at the end of the result stability time)	20 min	25 min

Other diagnostic tests (comparator, reference and confirmatory tests) provided for use in this study are:

- "WHO PQed RDT" Abbott SD Bioline HCV Test
- EIA 1: Diasorin Murex Anti-HCV 4.0
- EIA 2: Fujirebio INNOTEST HCV Ab IV
- LIA: Fujirebio INNO-LIA HCV Score
- HCV RNA assay (Abbott RealTime HCV assay for RNA PCR in Georgia; CE-IVD marked HCV RNA assay in Cambodia)
- HCV genotyping assay (Abbott RealTime HCV Genotype II assay in Georgia; Siemens Versant HCV genotype 2.0 in Cambodia - tbd)

Instructions for use of the investigational product(s) are provided separately from this protocol.

Medical device incidents, including those resulting from malfunctions of the device (or IVD), must be detected, documented and reported by the investigator at each site throughout the study (see section 7.1 and Appendix 2)

4.2 Preparation/Handling/Storage/Accountability

4.2.1 Acquisition

Procurement of the investigational products will be done through FIND, who will coordinate shipments from the manufacturer. It is the responsibility of each study site to maintain an updated inventory of the study materials and to inform FIND immediately if additional materials are required.

The investigator or designee must confirm appropriate temperature conditions have been maintained during transit for the investigational product received and any discrepancies are reported and resolved before its use.

4.2.2 Storage

Procedures for product storage and disposal will be described in the study manual.

The investigational product must be stored in a secure, environmentally controlled, and monitored (manual or automated) area in accordance with the labelled storage conditions, and access must be limited to the investigator and authorized site staff.

4.2.3 Test handling and performance

Testing using the investigational products will be performed according to the manufacturer’s instructions outlined in the study manual.

Only blood samples from participants enrolled in the study will be processed with the investigational product and only authorized site staff will be responsible for processing.

4.2.4 Accountability

The investigator is responsible for investigational product accountability, reconciliation, and record maintenance (i.e., receipt, reconciliation, and final disposition records). Investigational Product Accountability logs filled at each site will ensure the proper follow-up of the used, failed and remaining investigational products.

Further guidance and information for the final disposition of unused investigational product are provided in the study manual.

4.2.5 Export and import permits

It is expected that most countries will require import permits to receive the investigational materials. Local sites will be responsible for making import permit applications in a timely manner.

4.2.6 Quality control check for incoming shipments

Upon arrival of each new shipment of assays, the sites will conduct and document an incoming quality check following the study manual. New lots may only be used after this quality check is successfully passed.

4.2.7 Local procurement

Sites are responsible for assessing their needs and procuring any supplies, reagents and kits needed for the study that are locally available in order to include these costs in the study budget.

4.3 Minimisation of error and bias

4.3.1 Potential errors

There is a risk of procedural error when performing four different RDTs consecutively by one operator, as each test has slightly different procedures. This risk will be minimized by training the staff on the different RDT procedures as well as on how to process all four RDTs consecutively. Furthermore, the order of the investigational RDTs will be determined based on the required incubation time to ensure timings can be adhered to. The training will be performed on known HCV antibody negative and positive samples (de-identified samples provided by the reference laboratories), as well as fingerstick blood (procedural training only).

For each RDT, training on results reading will be conducted based on manufacturer-provided RDTs with bands of different intensities. If these cannot be provided by the manufacturers, training will be conducted on serial dilutions of known HCV antibody positive control samples.

All study samples and aliquots will be coded with unique FIND identifiers on pre-printed labels, to minimize the risk of sample mix-up due to mistakes in manual labelling

4.3.2 Potential bias

Participants will be enrolled sequentially as they present to the healthcare facility. Eligibility screening and consenting will be done by a person other than the healthcare professional who performs the test to avoid bias in RDT reading in case of a known HCV status. Patients with known HCV serology status will be instructed not to share this status with the HCV RDT tester.

Once the set enrolment targets for HCV negative individuals is reached, only individuals with known HCV-positive status will continue be enrolled in the study to meet required enrolment targets for HCV positive individuals. There is a risk to introduce bias in RDT interpretation, if all enrolled patients are known to be HCV positive. To minimise this risk, known HCV positive patients will be contacted and invited to participate in the study from the beginning of recruitment, to present to the facilities in the same timeframe as individuals of unknown, and thus often negative, HCV status.

The SD Bioline HCV RDT (WHO PQed) will be performed after the performance of the index tests to avoid interpretation bias by the reader based on the results of the WHO PQ-approved test.

EDTA plasma and serum samples will be tested freshly on all RDTs on the same day of sample collection to avoid difference in sample age compared to fingerstick whole blood from the same participant and between samples from different participants.

All reference tests will also be performed on fresh EDTA plasma samples within the allowed sample stability timeframe at 4°C (as per manufacturer’s IFU).

The laboratory technicians who perform reference testing, will not have access to the RDT results at the time of testing, as they will not require access to Open Clinica, where RDT results are

entered. This will minimize bias in LIA result interpretation, which is based on visual line intensity scoring. There is no bias in EIA result interpretation, as it is based on electronic read-outs (see section 10.2 Data management).

There is a risk of bias in LIA interpretation due to visual interpretation. This will be addressed by the scoring of all blots by two independent readers and joint discussion in case of discrepant interpretations. In case the two readers cannot agree, a third reader will be asked to score the results independently.

5 Participant discontinuation/withdrawal

5.1 Participant discontinuation/Withdrawal from the study

A participant may be withdrawn at any time, at the discretion of the investigator, for safety, behavioural, compliance, or administrative reasons.

If the participant withdraws consent for disclosure of future information, FIND may retain and continue to use any data collected before the withdrawal of consent.

If a participant withdraws from the study, he/she may request destruction of any samples taken and not tested, and the investigator must document this in the site study records. Participants who withdraw from the study will still have access to standard medical care.

5.2 Loss to follow up

No follow up visits are planned for study participants and as such there will be no incidents of loss to follow up.

6 Study procedures

Study procedures and their timing are summarized in the schedule of assessments (see Table 1) and outlined in Figure 2 below. Protocol waivers or exemptions are not allowed and adherence to the study design requirements, including those specified in the schedule of assessments, is essential and required for study conduct.

All screening evaluations must be completed and reviewed to confirm that participants meet all eligibility criteria. The investigator will maintain a screening log to record details of all participants screened and to confirm eligibility or record reasons for screening failure, as applicable.

A study laboratory manual will be compiled with all necessary details for on-site implementation and running of the study.

Figure 2*: Details of the study procedures, sample flow and timing of testing*

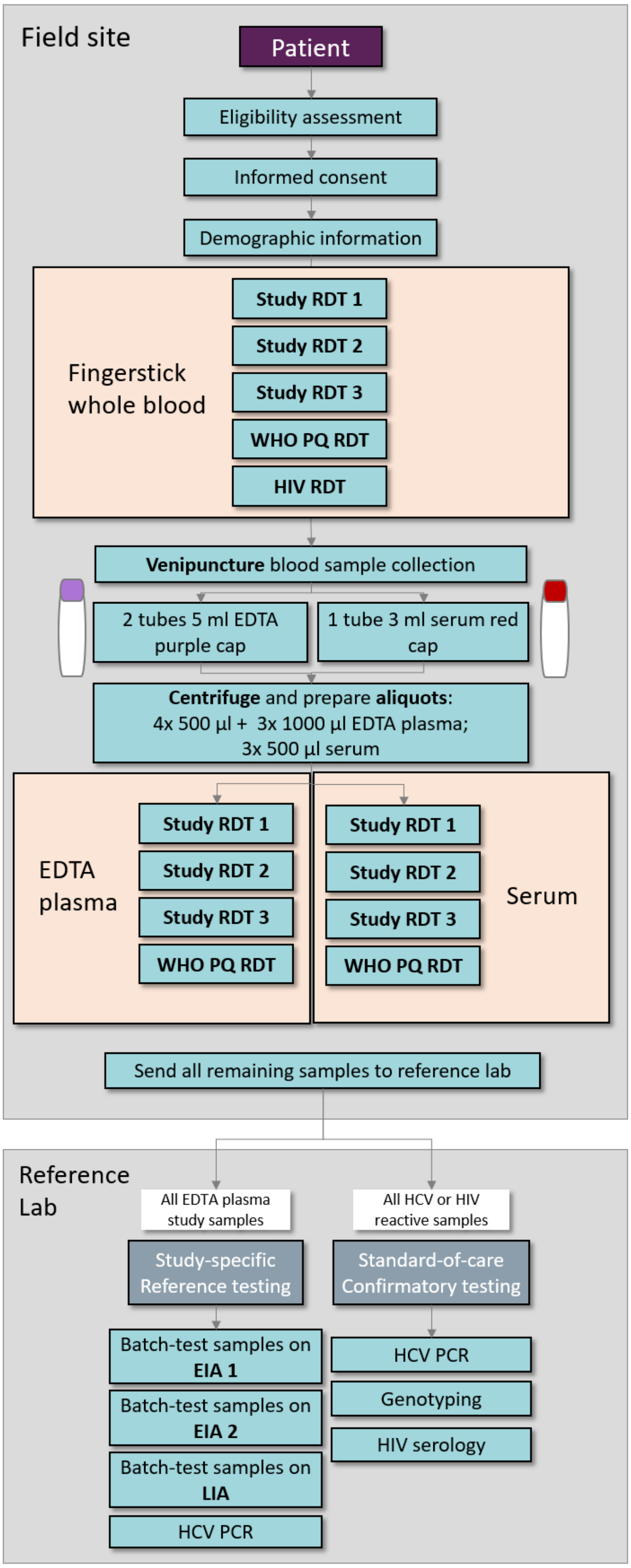

6.1 Participant enrolment

Enrolment will be done at primary healthcare facilities by trained staff.

All individuals attending the healthcare facilities for any health-related issue will be invited to participate in the study.

Individuals will be screened for eligibility and must provide their written consent to participate. After obtaining the signed consent, a participant ID is assigned to him/her and the participant is recorded in the enrolment log.

A participant who is not yet registered at the local healthcare facility, will be entered into care through the locally required registration procedure.

At enrolment, the following information will be collected for each participant, based on verbal information provided by the individual:

- Age
- Gender
- HCV status (known/unknown)
- Medications taken (including antiretrovirals)
- Recent vaccination (within the last 12 months)
- Concomitant infections (including HIV and approximate date of last negative HIV test, if applicable)

After recording the participant’s demographic and clinical data in the dedicated Participant Worksheet, blood samples can be collected to perform all necessary study testing.

6.2 RDT testing

6.2.1 Fingerstick blood

Fingerstick blood will be collected according to each manufacturer’s IFU, e.g. with a provided pipette or capillary. All RDTs for a single participant will be performed by the same operator, who is a trained healthcare worker at the facility. The three selected study RDTs have an incubation time of 10-20 min prior to result readiness, which will allow for all RDTs to be started consecutively and read more or less simultaneously, once the incubation time is over and within the allowed reading time frame (see Table 3).

Results of the study RDTs will be read before results of the WHO PQed RDT and all results will be recorded by the operator into the Participant Worksheet.

Any invalid RDT runs will be repeated immediately, using a fresh drop of fingerstick blood, EDTA plasma or serum.

6.3 Venous blood draw and aliquot preparation

Once the fingerstick whole blood collection procedure is completed, the participant will proceed for a venous blood draw of 2 x 5 ml whole blood in an EDTA plasma tube and 1x 3 ml whole blood in a serum tube. All tubes will be pre-labelled with a study specific ID.

After the appropriate clotting time for the serum, all tubes will be centrifuged at a time and speed indicated by the tube manufacturer.

Plasma samples will subsequently be aliquoted in 3x 1 ml aliquots (for HCV RNA PCR, genotyping and potential repeat testing) and 4x 0.5 ml aliquots for RDT and reference testing. Serum samples will be aliquoted in 3x 0.5 ml aliquots for RDT testing.

After aliquot preparation, RDT testing on the EDTA plasma and serum samples will be performed. All other samples will be stored at 4°C until cooled transportation to the reference laboratory. Samples can be stored at 4°C for up to 7 days until EIA and LIA testing, according to the sample stability provided in the manufacturer's IFU [21-23].

All left-over samples will be stored at the reference laboratories at -70°C until the study is completed. Once the study is completed, the samples will be transferred to the FIND sample repository at ZeptoMetrix (for storage time and intended use of left-over samples, see section 6.5).

6.4 Reference and confirmatory testing

After transportation to the reference laboratory, all samples will be stored at 4°C until EIA1, EIA2 and LIA reference testing, and HCV and HIV confirmatory testing, as well as HCV genotyping. All procedures will be performed within the allowed sample stability timeframe for 4°C storage by the manufacturer.

EIA1, EIA2 and LIA reference testing on EDTA plasma samples will be performed in batches. Performance of these test is known to be equivalent in serum and EDTA plasma, according to the manufacturers' IFU. Due to this, reference testing will only be performed in EDTA plasma. For each EIA run, the manufacturers' positive and negative controls will be used and results interpreted according to the IFU. Additionally, a plate-internal run control (IRC) of a known HCV-positive sample, will be added to each EIA plate, to monitor EIA consistency. The expected value of this IRC will be set during three independent control runs prior to the start of any EIA reference testing. IRC standard deviations and acceptance limits will be set based on the IRC results from these three control runs. Further details will be provided in the study manual.

6.5 Specimen collection, handling, transport and storage

All samples will be handled in accordance with standard local biosafety rules and maintained at 4°C for transportation.

The maximum amount of blood collected from each participant over the duration of the study, will not exceed 13 ml of venous blood and 4 drops of fingerstick blood (approximately 50 µl each). Repeated or unscheduled samples may be taken for safety reasons or for technical issues with the samples.

The fingerstick blood samples will be used immediately on the RDTs. The 13 ml venous blood will be collected in 2x 5 ml EDTA tubes and a 1x 3 ml serum tube. Plasma and serum will be separated by centrifugation within a maximum of 4 hours from blood collection at the healthcare facilities. The plasma and serum aliquots will be stored at 4°C until transfer to the reference lab for EIA/LIA, reference and confirmatory testing.

Left-over samples will be archived at the ZeptoMetrix biorepository for a maximum of 5 years or until FIND agrees that samples can be destroyed. For sample destruction (e.g. upon withdrawal of consent to use blood samples), best practices for management of infectious waste should be followed.

6.6 Index test and reference standard test procedures

Test procedures for the reference tests and the RDT index tests will be performed according to the manufacturer's IFU, which are provided separately from this protocol.

6.6.1 Index tests procedure summary

Wantai RDT:	one drop of whole blood or 50 µl of serum/plasma, 1 drop of buffer, 10 min incubation time
PMC RDT:	one drop of whole blood or 35 µl of serum/plasma, 1 drop of buffer, 20 min incubation time
AccessBio: RDT	10 µl of whole blood, serum or plasma, 2 drops of buffer, 15 min incubation time

The fingerstick whole blood sample will be applied with a small pipette provided with the kit and according to the manufacturer's instructions. Plasma and serum samples will also be applied with a small pipette, which the manufacturers will provide additionally, as each test only includes one pipette. All RDTs contain a procedural/sample control line to check and verify that sample has been applied and the procedure was performed correctly.

6.6.2 Reference tests for composite reference standard

- EIA 1: Diasorin Murex Anti-HCV 4.0
- EIA 2: Fujirebio INNOTEST HCV Ab IV
- LIA: Fujirebio INNO-LIA HCV Score

The composite reference standard is designed of two EIAs and one LIA.

While EIAs have overall good performance characteristics [21, 22], the LIA is intended to serve as a more specific supplemental tests in samples reactive by EIA [23]. In addition to high specificity, the LIA has very good sensitivity. Both performance characteristics are owed to the presence of individual recombinant antigen lines on the LIA membrane.

Due to the intended superior performance characteristics of LIA to EIAs, any samples which have discrepant results in the EIAs to the LIA will be excluded from the main analysis of the study (i.e. 2x positive EIA and negative LIA or 2x negative EIA and positive LIA) to minimize the risk of false-negatives or false-positives results from the composite reference standard. In case of a discrepant results in the EIA, the LIA is the decision-making test result (see also Table 8).

This algorithm is used by WHO PQ for sample characterisation during the independent laboratory evaluations of RDTs [17], with the exception that LIA is not performed on samples which are negative with both EIAs. For the present study, it was decided that all samples will be tested by LIA, like in the previous evaluation of HCV RDT led by FIND [4]. In this study, a small subset of samples negative in both EIAs was positive or indeterminate in LIA, suggesting the presence of HCV antibodies.

Further combinations of the reference results will be used to assess tests performance in borderline and more difficult samples (for further details see section 8.4.1).

6.6.3 Discordant results

Discordant results of the study RDTs to the WHO PQed RDT (SD Bioline) will be repeated for EDTA plasma and serum samples only, as the participant may not be available for repeat testing of fingerstick whole blood. Discordant results will be repeated in duplicate within 24h at the primary healthcare facility, using the same lot.

Performance analysis will be conducted using initial results only. The results of further testing of samples with discrepant results will be reported separately as additional information about RDT performance.

6.6.4 Training

All healthcare workers at the enrolment sites will be trained in the procedures of the study RDTs, including workflow of test reading and result recording. Training will be performed on known positive and negative plasma or serum samples, as well as on fingerstick whole blood. Interpretation of RDT bands will be trained on pre-conceived RDTs provided by the manufacturers or obtained by serial dilutions of known positive samples. Proficiency will be documented in a training log.

Training on the reference tests will be restricted to reviewing the IFU as the chosen laboratories are familiar with the performance of EIAs and LIA. Prior to starting sample testing on EIA, a positive IRC needs to be adjusted to fall within the low cut-off range of the specific EIAs. This adjustment testing can serve as training runs in case the laboratory technicians are not familiar with the exact procedure of the study-specific EIAs. Familiarization of the test procedures will be documented in a training log.

6.7 Confirmatory testing and genotyping

All participants with an initially reactive RDT will have an HCV viral load assessment and the HCV genotype assessed in case of sufficiently high viral load (depending on the genotyping kit's IFU on minimum needed RNA). See section 4.1 for specific products. No genetic analysis will be performed on human genetic material.

6.8 Experimental Biomarkers

Left-over samples may be used for research to develop or evaluate methods, assays, prognostics and/or companion diagnostics related to other infectious viral hepatitis diseases or potentially frequently found co-infections in affected target populations (such as HIV or TB).

Samples will be stored for a maximum of 5 years at the ZeptoMetrix biorepository (or until FIND agrees that samples can be destroyed), following the last participant's sample collection for the study. The participants will be asked to provide a separate informed consent for further sample analysis (see also section 9.3 Informed consent process).

6.9 Other tests

Every study participant will receive an HIV test, as HIV infection may influence HCV RDT test performance due to lower antibody titres in immunocompromised patients [19, 20]. This may allow to draw conclusions on false negative RDT results.

Reactive HIV RDT results will be followed up with the standard of care confirmatory test. The results will be communicated to the participant through the participant's physician. All enrolled participants have consented to the performance of an HIV test, which will be performed if a positive HIV status is not documented in their medical record or the last negative HIV test result is older than 12 months.

6.10 Safety assessments

There will be no planned safety assessments as the risks for the participants are considered minimal (see section 1.3).

6.11 Other study procedures

A technical appraisal of each RDT product per manufacturer will be completed at the end of the study jointly by all healthcare workers who have performed this test. The appraisal will be documented in the "RDT Technical Appraisal Form" (see Appendix 3). Each site completes one technical appraisal form per manufacturer.

7 Safety and incident reporting

Given that this is a diagnostic accuracy study of HCV RDT assays, the probability of an adverse event (AE) or a serious adverse event (SAE) occurring to a study participant to be associated with the investigational products is extremely low (see Appendix 1 for definitions of AE or SAE). Considering this, only deaths occurring between the start of the fingerstick blood collection and until the participant leaves the study will be recorded on a standardized form and reported to FIND by the investigator within 24 hours.

7.1 Medical device incidents (including malfunctions)

Medical devices are being provided for use in this study for the purpose of evaluating HCV RDT diagnostic accuracy. In order to fulfil regulatory reporting obligations worldwide, the investigator is responsible for the detection and documentation of events meeting the definitions of incident or malfunction that occur during the study with such devices. The definition of a medical device incident can be found in Appendix 2.

Examples of a medical device incident for this study could be:

- A broken safety lancet provided in the RDT kit (if provided)
- A broken or malfunctioning capillary tube or pipette provided in the RDT kit

NOTE: Incidents fulfilling the definition of an SAE will also follow the processes outlined above and in Appendix 2 of the protocol.

7.1.1 Time period for detecting medical device incidents

Medical device incidents or malfunctions of the device that result in an incident will be detected, documented, and reported during all periods of the study in which the medical device is used.

If the investigator learns of any incident at any time after a participant has been discharged from the study, and such incident is considered reasonably related to a medical device provided for the study, the investigator will promptly notify FIND.

The method of documenting medical device incidents is provided in Appendix 2.

7.1.2 Follow-up of medical device incidents

All medical device incidents involving a SAE will be followed up and reported in the same manner as other SAEs. This applies to all participants, including those who discontinue study intervention.

The investigator is responsible for ensuring that follow-up includes any supplemental investigations as indicated to elucidate the nature and/or causality of the incident.

New or updated information will be recorded on the originally completed form with all changes signed and dated by the investigator.

7.1.3 Reporting of medical device incidents to FIND

Medical device incidents will be reported to FIND within 24 hours after the investigator determines that the event meets the protocol definition of a medical device incident.

The Medical Device Incident Report Form will be sent to FIND by email. If email is not available, a mobile phone picture can be taken and sent via messaging service.

The same individual(s) will be the contact for the receipt of medical device incident reports and SAE reports.

7.1.4 Regulatory reporting requirements for medical device incidents

The investigator will promptly report all incidents occurring with any medical device provided for use in the study in order for FIND to fulfil the legal responsibility to notify appropriate regulatory authorities and other entities about certain safety information relating to medical devices being used in clinical studies.

The investigator, or responsible person according to local requirements (e.g., the head of the medical institution), will comply with the applicable local regulatory requirements relating to the reporting of incidents to the IRB/IEC.

8 Statistical considerations

8.1 Statistical hypotheses

No statistical hypotheses will be tested in this study.

8.2 Sample size determination

For the three RDTs – Wantai, PMC and AccessBio – the previous FIND HCV RDT study [4] estimated an overall sensitivity of 97.1%, 95.9% and 96.1%, and an overall specificity of 97.5%, 99.5% and 97.8% in EDTA plasma, respectively (unpublished data).

Considering the possibility of a lower performance in fingerstick whole blood in primary healthcare settings, the sensitivity and specificity for these RDTs in the present study was conservatively assumed to be 90%. For fresh EDTA plasma and serum, sensitivity and specificity were assumed to be similar to the previous study, i.e. at least 95%.

Considering that the study aim is to obtain point estimates of sensitivity and specificity and positive and negative predictive values for each test, the assumed values of 90% performance in whole blood and 95% performance in EDTA plasma/serum were used as reference to calculate the sample size so that the study would have 80% power to obtain the estimates with a precision of ±5%. The minimum sample size required was determined following the methods described in "Statistical Methods in Diagnostic Medicine", chapter 6 [24].

The study sample was increased from the statistically required minimum in order to meet the requirements in the WHO Technical Specification Series document "TSS-7: Rapid diagnostic tests to detect hepatitis C antibody or antigen" (still in draft) [6]. The WHO TSS-7 requires at least 400 HCV antibody positive samples to be evaluated for sensitivity in fresh whole blood, and at least 1'000 HCV antibody and HCV RNA negative samples for specificity in fresh whole blood. An additional 10% was added to the required sample size to account for sample exclusion due to non-confirmed antibody status based on the composite reference standard (see ***Error! Reference source not found.***). This number was based on experience from the previous FIND HCV RDT study, which used the same composite reference standard [4].

Table 4 reports the minimum sample size requirements calculated and the final sample size for this study.

Table 4: *Sample size*

		**Estimated performance**	**Power**	**Margin**	**Statistical sample size requirement**	**WHO TSS-7 sample size requirement**	**Final sample size for this study**
Fingerstick whole blood	Sensitivity	90%	0.8	0.05	283	400	**440**
	Specificity	90%	0.8	0.05	283	1'000	**1'100**
EDTA plasma	Sensitivity	95%	0.8	0.05	150	400	**440**
	Specificity	95%	0.8	0.05	150	1'000	1'100
Serum	Sensitivity	95%	0.8	0.05	150	400	**440**
	Specificity	95%	0.8	0.05	150	1'000	**1'100**

Each participant will provide samples for all sample types (see also Figure 2), resulting in a total of 1'540 matched fingerstick whole blood, EDTA plasma and serum sample sets.

Enrolment targets per country and site are listed in **Table 5**. Targets take into consideration local HCV prevalence and estimated number of patients to be enrolled per day

Table 5 *Enrolment targets per country and site*

<table>
<tr><th></th><th>Approximate HCV prevalence</th><th>Estimated number of patients recruited per day</th><th>HCV antibody positive participants</th><th>HCV antibody/RNA negative participants</th><th colspan="2">Total</th></tr>
<tr><td>Georgia OST site</td><td>40% + patients with known HCV status</td><td>25</td><td>310</td><td>140</td><td>450 per site</td><td rowspan="2">770 per country</td></tr>
<tr><td>Georgia NCDC screening site</td><td>5%</td><td>15</td><td>20</td><td>300</td><td>320 per site</td></tr>
<tr><td>SHCH outpatient site</td><td>5% + patients with known HCV status</td><td>25</td><td>110</td><td>660</td><td colspan="2">770 per site and country</td></tr>
</table>

8.3 Populations for analyses

For purposes of analysis, the following populations will be defined as per **Table 6**.

Table 6*: Population for analyses*

Population	Description
Enrolled	All participants who sign the informed consent
Evaluable/Per Protocol Population (PP)	All participants who fully complied with the protocol and for whom index test results and reference test results are available for all tests.
Partially Compliant Population (PCP)	All participants who complied partially with the protocol, i.e. those for whom index test results and reference test results are available but not in a complete form (e.g. missing one RDT result)

8.4 Statistical analysis plan

The statistical analysis plan (SAP) will be developed and finalized before the start of enrolment and will describe the analysis strategy in detail. This section is a summary of the planned statistical analyses of the primary and secondary endpoints.

8.4.1 Analysis of primary and secondary outcomes

Estimates of sensitivity, specificity, positive and negative predictive values, together with 95% confidence intervals based on Wilson's method, will be obtained based on the definitions reported in Table 7. The estimates will be obtained for each RDT separately, using either the composite reference standard or the WHO PQed HCV RDT (SD Bioline) as reference standard for the outcomes 1.1 and 2.1 respectively. Outcomes 1.1 and 2.1 will be evaluated on the PP population.

Table 7 outlines the classification of sample results for calculation of sensitivity, specificity, PPV and NPV.

***Table 7**: Definition of test results and classification metrics*

	Composite reference standard outcome			
**RDT result**		HCV antibody positive	HCV antibody negative	Total
	HCV antibodies detected	a	b	a+b
	HCV antibodies not detected	c	d	c+d
	Total	a+c	b+d	a+b+c+d

<u>Definitions and calculation:</u>

a = True Positives, b = False Positives

c = False Negatives, d = True Negatives

Sensitivity = a / (a + c)

Specificity = d / (b + d)

Positive Predictive value = a / (a + b)

Negative Predictive value = d / (c + d)

Point estimates (with 95% CI) of sensitivity, specificity, PPV and NPV will derived separately for each site, as well as for the pooled data for all sites. In order to assess whether there is an association between the results and the clinical site from where the samples were collected, the estimates of sensitivity and specificity for each individual site will be compared with each other by use of a Pearson’s chi-squared test, at a significance level of 5%, adjusted by Bonferroni correction for the total number of tests performed. A random effect model will be used if it is believed that a high site-effect is present: this will be assessed by the evaluation of heterogeneity using Cochran’s Q (with a significance level set at 0.1) and the I^2 statistics (with a value >0.5).

Table 8 outlines the combinations of the reference method results and the corresponding composite reference standard outcomes. All EIA and LIA result are interpreted according to the manufacturers IFU (pos, neg, indet). See section 6.6.2 for a further explanation of the composite reference standard.

***Table 8**: Reference method results and corresponding composite reference standard outcomes*

		Composite reference standard outcome											
		HCV antibody positive			HCV antibody negative			Sample excluded					
**Reference method results**	**EIA1**	Pos	Pos	Neg	Neg	Neg	Pos	Pos	Neg	Pos	Pos	Neg	Neg
	**EIA2**	Pos	Neg	Pos	Neg	Pos	Neg	Pos	Neg	Pos	Neg	Pos	Neg
	**LIA**	Pos	Pos	Pos	Neg	Neg	Neg	Neg	Pos	Indet	Indet	Indet	Indet

Additional subgroup analyses will be performed for different combinations of reference method outcomes, further detail will be provided in the statistical analysis plan.

The details on the analysis of outcome 2.2 will be described in the study SAP.

8.4.2 Efficacy analysis

The efficacy of the index test to measure true positive or true negative HCV antibody serology status is expressed in the calculated sensitivity and specificity.

8.4.3 Safety analysis

No safety analysis will be performed as the risks for the participants are considered minimal (see section 1.3).

8.4.4 Other analyses

Descriptive statistics tables will be generated to summarize the characteristics of the samples in the different populations. The number of samples included and excluded will be reported, and among the included samples, information will be broken down by site, gender, age group, HCV, HIV status and HCV genotype. Results will be reported either in absolute numbers (e.g. number of subjects in a group) or summarized by mean, standard deviation, minimum, median, maximum and quartiles.

Information on HCV RNA viral load, as well as HIV status, may be used for interpretation of HCV RDT performance.

A sub-analysis on the objectives for the individual study countries will also be performed.

8.5 Planned interim analyses

No interim analyses are planned for this study.

8.5.1 Data Monitoring Committee (DMC)

There will be no data monitoring committee. Interim data monitoring will be conducted by FIND.

9 Regulatory and ethical considerations

9.1 Regulatory and ethics approvals

This study will be conducted in accordance with the protocol and with the following:

- Consensus ethical principles derived from international guidelines including the Declaration of Helsinki
- Applicable Good Clinical Practice Guidelines: ICH GCP E6 (R2)
- Applicable laws and regulations

The protocol, protocol amendments, Informed Consent Forms (ICF) and other relevant documents (e.g. advertisements) must be submitted to an Institutional Review Board/Independent Ethics Committee (IRB/IEC) by the investigator and reviewed and approved by the IRB/IEC before the study is initiated. A copy of the IRB/IEC approval letter will be filed in the investigator site file.

FIND-approved versions of an amended study protocol must be signed by the investigator(s). Any substantial amendments to the protocol will require IRB/IEC approval before implementation of changes made to the study design, except for changes necessary to eliminate an immediate hazard to study participants. Protocol amendments restricted to clerical edits only will be provided to the study sites and submitted to the IRB/IEC for informational purposes.

The investigator will be responsible for the following:

- Providing written summaries of the status of the study to the IRB/IEC annually or more frequently in accordance with the requirements, policies, and procedures established by the IRB/IEC
- Notifying the IRB/IEC of SAEs or other significant safety findings as required by IRB/IEC procedures
- Providing oversight of the conduct of the study at the site and adherence to requirements of ICH GCP guidelines, the IRB/IEC, the WHO Good Clinical Laboratory Practice (GCLP), and with applicable national regulations.

9.2 Financial disclosure

Investigators and sub-investigators will provide FIND with sufficient, accurate financial information as requested to allow FIND to submit complete and accurate financial certification or disclosure statements to the appropriate regulatory authorities.

9.3 Informed consent process

The investigator or his/her representative will explain the nature of the study to the participant in a language understandable to him/her and answer all questions about the study.

Participants must be informed that their participation is voluntary. Participants will be required to sign and date a statement of informed consent that meets the requirements of the ICH GCP E6 R2 guidelines where applicable, and of the IRB/IEC or study centre.

There must be evidence that written informed consent was obtained before the participant was enrolled in the study, and ample time was given to participant to consent. The date the written consent was obtained (as well as the time, ideally) must be recorded. The authorised person obtaining the informed consent must also sign and date the ICF.

Illiterate participants must provide a thumbprint on the ICF and the ICF must be signed and dated by an impartial witness.

Participants must re-consent to the most current version of the ICF(s) during their participation in the study.

A copy of the ICF(s) will be given to the participant.

Participants who are rescreened are required to sign a new ICF.

The ICF will contain a separate section that addresses the use of left-over samples for optional exploratory research. The investigator or authorized designee will explain to each participant the objectives of the exploratory research. Participants will be told that they are free to refuse to participate and may withdraw their consent at any time and for any reason during the storage period. A separate signature will be required to document a participant's agreement to allow any remaining specimens to be used for exploratory research. Participants who decline to participate in this optional research will not provide this separate signature.

9.4 Data protection

Participants will be assigned a unique identifier generated by FIND. Any participant records or datasets that are transferred to FIND will contain the identifier only; participant names or any information which would make the participant identifiable will not be transferred.

The participant will be informed that his/her personal study-related data will be used by FIND in accordance with local data protection law. The level of disclosure must also be explained to the participant.

The participant will be informed that his/her medical records may be examined by quality assurance auditors or other authorized personnel appointed by FIND, by appropriate IRB/IEC members, and by inspectors from regulatory authorities.

A generic example of a FIND participant personal identifier (PPID) is shown below.

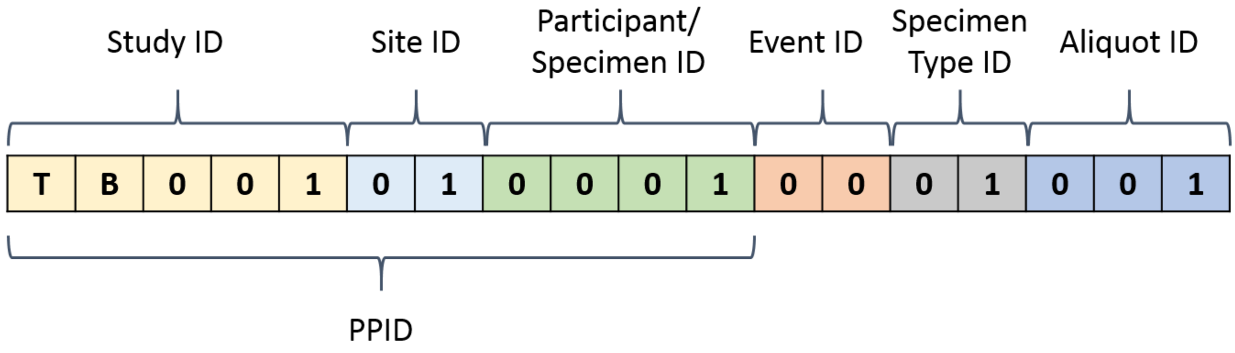

10 Data handling and record keeping

FIND is responsible for the data management of this study, including quality control checks of the data and assessment of overall protocol compliance. All participant data relating to the study will either be directly entered into Open Clinica (OpenClinica Enterprise Edition *version 4.0*) or be recorded on paper or electronic source documents and transcribed into Open Clinica or electronic Result Entry Forms for upload to Open Clinica (see Figure 3 in section 10.2 Data Management). The investigator is responsible for verifying that data entries are accurate and correct by electronically signing the CRF.

Records and documents, including signed ICFs, pertaining to the conduct of this study must be retained by the investigator for 10 years after study completion unless local regulations or institutional policies require a longer retention period. No records may be destroyed during the retention period without the written approval of FIND. No records may be transferred to another location or party without written notification to FIND.

FIND's archive policy, in accordance with Swiss Law, is 10 years.

10.1 Source data and source documents

Source documents provide evidence for the existence of the participant and substantiate the integrity of the data collected. The investigator/institution should maintain adequate and accurate source documents and study records that include all pertinent observations on each of the site's study participants (source data). The investigator may need to request previous medical records or reports (if available), depending on the study. Source documents are filed at the investigator's site.

Source data should be attributable, legible, contemporaneous, original, accurate and complete. Changes to source data should be traceable, should not obscure the original entry and should be explained, if necessary.

The investigator must permit study-related monitoring, audits, IRB/IEC review, and regulatory agency inspections and provide direct access to participant medical records and source documents used for this study.

For the purpose of this study, the source data are defined as shown in Figure 3 below.

The definition of the source data will further be refined and documented during the site initiation visit.

10.2 Data management

Data management procedures at FIND, including the setup of the database, programming edit and querying, are described in the Data Management Plan.

Whenever possible, clinical data and laboratory results will be captured directly onto electronic CRFs designed by FIND in the Open Clinica Enterprise version 4 EDC system. Site staff will be responsible for entering their data from a paper-based Participant Worksheet into Open Clinica where direct capture into EDC is not possible. Detailed timelines for data transfer will be provided in the study manual. Data will be cleaned of errors by FIND throughout the study as it is captured electronically.

The investigator is responsible for verifying that data entries are accurate and correct. Data entered in the Open Clinica database must be consistent with the source documents or the discrepancies must be explained.

The site will be provided with individual password-protected accounts to access Open Clinica, following a training session given by FIND.

Open Clinica provides an audit trail system recording all data entries/changes and queries between FIND and the site. Data entry training will be provided by FIND, either on site or remotely sharing screen through Skype or any other similar system.

Figure 3*: Data management concept and source data description*

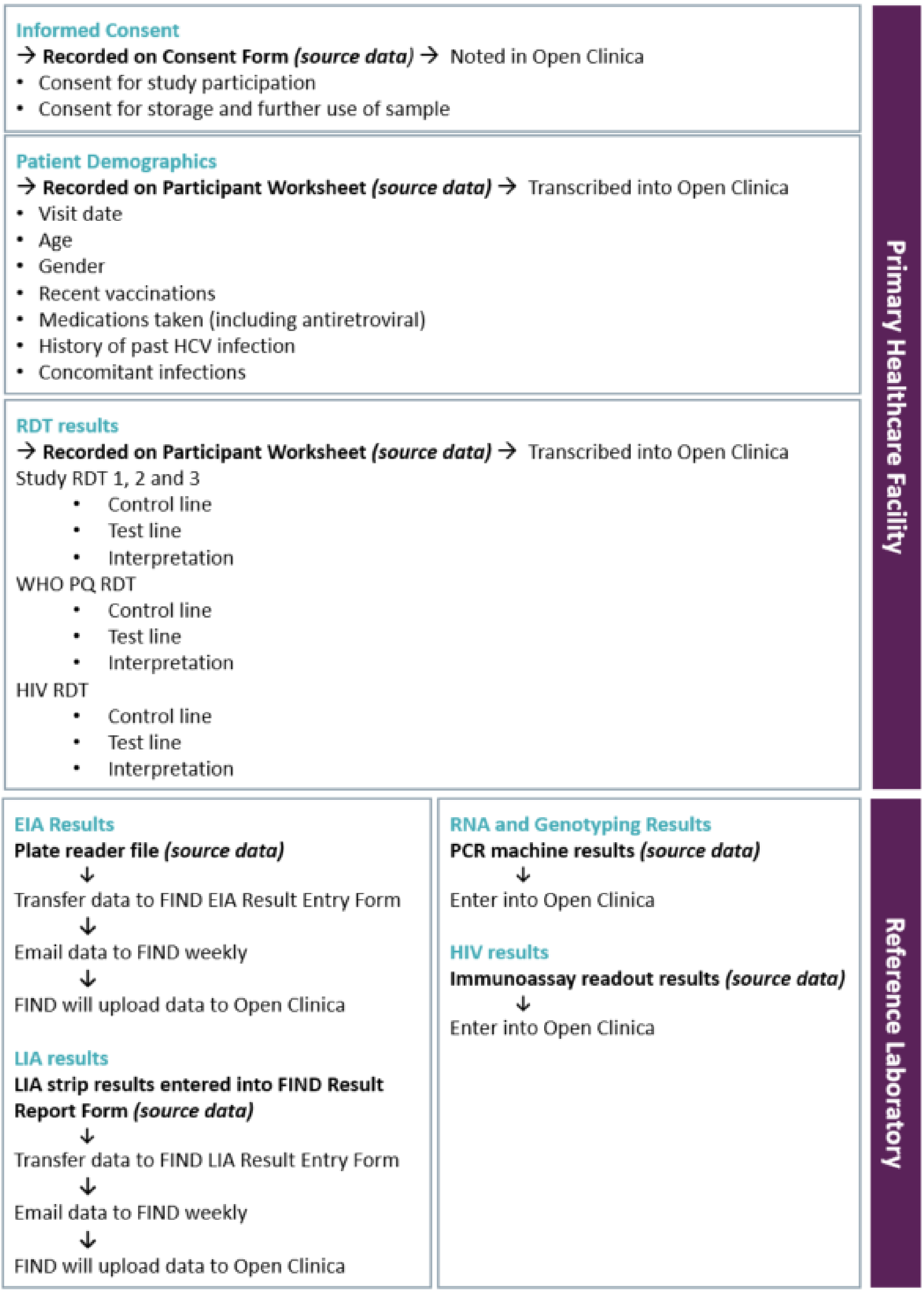

No information concerning the study or the data generated from the study will be released to any unauthorized third party without prior written approval of FIND.

11 Quality management

Quality Management for this study consists of Quality Control activities, training and capacity building provided by FIND (or designee) to the investigational sites and laboratories, as well as the use of standard operating procedures, work instructions, tools and templates.

Training on the protocol, GCP and the use of the IVDs and laboratory tests will be provided by FIND. A Study Laboratory Manual, which describes all of the sample testing procedures, will be provided by FIND prior to the commencement of the study. Training on the EDC system will be provided by FIND Data Management prior to first participant enrolment.

11.1 Quality control (monitoring)

Quality control should be applied to each stage of data handling to ensure that all data are reliable and have been processed correctly. The investigational site is responsible for performing regular quality control checks on the data they generate.

FIND will perform risk based monitoring of this study, and associated quality control checks, as described in the Monitoring Plan. Study monitors will perform source data review and source data verification to confirm that data entered into the CRF by authorized site personnel are accurate, complete, and verifiable from source documents; that the safety and rights of participants are being protected; and that the study is being conducted in accordance with the currently approved protocol and any other study agreements, ICH GCP, and all applicable regulatory requirements.

11.2 Quality assurance (auditing)

As part of routine quality assurance, FIND or designee may conduct an audit of the investigational site.

The Lugar Centre and the Institut Pasteur du Cambodge are ISO 15189 accredited laboratories and participate in regular external quality assurance assessments.

11.3 Study and site closure

FIND reserves the right to close the study site or terminate the study at any time for any reason at its sole discretion. Investigational sites will be closed upon study completion. A study site is considered closed when all required documents and study supplies have been collected and participant samples shipped to the FIND biorepository.

The investigator may initiate study-site closure at any time, provided there is reasonable cause and sufficient notice is given in advance of the intended termination.

Reasons for early closure of a study site by FIND are described in the contractual agreement.

12 Publication policy

Data obtained from participation in this study are considered confidential and may be used to support market approval. The investigators must adhere to the non-disclosure requirements set forth in the FIND agreement.

The investigator is obligated to provide FIND or its designee with complete test results and all data obtained in this study. Only FIND may make information obtained during this study available to other investigators and third parties.

Authorship for scientific publication of the study results will be determined by mutual agreement and in line with International Committee of Medical Journal Editors authorship requirements, as described in the publication policy section of the contractual agreement.

13 References

1. WHO. *World Health Organization: Guidelines on Hepatitis B and C Testing*. 2017 21/06/2018]; Available from: http://apps.who.int/iris/bitstream/handle/10665/254621/9789241549981-eng.pdf;jsessionid=ECE9BAB91A029E9A9F76E0E92585ADE6?sequence=1.
2. WHO. *World Health Organization: In vitro diagnostics and laboratory technology: Simple/Rapid tests*. 2018 02. July 2018]; Available from: http://www.who.int/diagnostics_laboratory/faq/simple_rapid_tests/en/.
3. WHO, *World Health Organization: Overiew of the WHO prequalification of in vitro diagnostics assessment* https://apps.who.int/iris/bitstream/handle/10665/259403/WHO-EMP-RHT-PQT-2017.02-eng.pdf;jsessionid=387D24F53BDCAA88F4DBA8BFD1B8DBF1?sequence=1, 2018.
4. FIND, *Evaluation study of Rapid Diagnostic Tests (RDTs) detecting antibodies against hepatitis C virus; protocol 8162-2/1 v3.0*, in *Clinical Trial Protocol*. 2018.
5. WHO. *World Health Organization: Selecting and purchasing HIV, HBsAg and HCV in vitro diagnostics*. 2018 [cited 2019 15-Feb].
6. WHO. *World Health Organization: TSS-7 Rapid diagnostic tests to detect hepatitis C antibody or antigen (Draft)*. 2019 30-Jan-2019]; Available from: https://www.who.int/diagnostics_laboratory/guidance/draft_tss7_rdts_to_detect_hepatitis_c_antibody_antigen.pdf?ua=1.
7. WHO. *World Health Organization: Global Hepatitis Report 2017*. 2017 02. July 2018]; Available from: http://apps.who.int/iris/bitstream/10665/255016/1/9789241565455-eng.pdf?ua=1.
8. Pawlotsky, J.M., *New hepatitis C therapies: the toolbox, strategies, and challenges.* Gastroenterology, 2014. **146**(5): p. 1176-92.
9. Feeney, E.R. and R.T. Chung, *Antiviral treatment of hepatitis C.* BMJ, 2014. **348**: p. g3308.
10. WHO. *World Health Organization: Guidelines for the Screening, Care and Treatment of Persons with Chronic Hepatitis C Infection* 2016 21/06/2018]; Available from: http://apps.who.int/iris/bitstream/handle/10665/205035/9789241549615_eng.pdf?sequence=1.
11. D. Baliashvili, A.K., G. Kuchukhidze, S. Salyer, A. Gamkrelidze, K. Zakhashvili, M. Alkhazashvili, G. Chanturia, N. Chitadze, R. Sukhiashvili, M. Shakhnazarova, P. Imnadze, J. Drobeniuc, C. Blanton, S. Russell, J. Morgan, F. Averhoff, L. Hagan, *Prevalence and genotype distribution of hepatitis C virus in Georgia: a 2015 nationwide population-based survey.* Journal of Hepatology, 2017. **66**(1): p. Supplement S277.
12. De Weggheleire, A., et al., *A cross-sectional study of hepatitis C among people living with HIV in Cambodia: Prevalence, risk factors, and potential for targeted screening.* PLoS One, 2017. **12**(8): p. e0183530.
13. Nguyen, L.H. and M.H. Nguyen, *Systematic review: Asian patients with chronic hepatitis C infection.* Aliment Pharmacol Ther, 2013. **37**(10): p. 921-36.
14. Reipold, E.I., et al., *Values, preferences and current hepatitis B and C testing practices in low- and middle-income countries: results of a survey of end users and implementers.* BMC Infect Dis, 2017. **17**(Suppl 1): p. 702.
15. Tang, W., et al., *Diagnostic accuracy of tests to detect Hepatitis C antibody: a meta-analysis and review of the literature.* BMC Infect Dis, 2017: p. 695.

16. Chevaliez, S., et al., *Prospective assessment of rapid diagnostic tests for the detection of antibodies to hepatitis C virus, a tool for improving access to care.* Clin Microbiol Infect, 2016: p. 459 e1-6.
17. WHO. *World Health Organization: WHO PROTOCOL FOR PERFORMANCE LABORATORY EVALUATION OF HCV SEROLOGY ASSAYS*. WHO PQDX_040 v6 February 2017 2017 [cited 2019 15-Feb].
18. Robin, L., et al., *Analytical performances of simultaneous detection of HIV-1, HIV-2 and hepatitis C- specific antibodies and hepatitis B surface antigen (HBsAg) by multiplex immunochromatographic rapid test with serum samples: A cross-sectional study.* J Virol Methods, 2018. **253**: p. 1-4.
19. Chamot, E., et al., *Loss of antibodies against hepatitis C virus in HIV-seropositive intravenous drug users.* AIDS, 1990. **4**(12): p. 1275-7.
20. Juniastuti, et al., *High rate of seronegative HCV infection in HIV-positive patients.* Biomed Rep, 2014. **2**(1): p. 79-84.
21. Diasorin. *Murex anti-HCV (version 4.0) product specifications*. 2018 03-July-2018]; Available from: https://www.diasorin.com/sites/default/files/allegati_prodotti/Murex%20Anti-HCV%20Version%204.0.pdf.
22. Fujirebio, *INNOTEST HCV Ab IV Instructions for Use*. 2014.
23. MPBIO. *HCV Blot 3.0 Product Description*. 2018 02.July 2018]; Available from: https://www.mpbio.com/product.php?pid=0711130018&country=81.
24. Zhou, X.-h., D.K. McClish, and N.A. Obuchowski, *Statistical methods in diagnostic medicine*. 2nd ed. Wiley series in probability and statistics. 2011, Hoboken, N.J.: Wiley. xxx, 545 p.

14 Appendices

Appendix 1: Safety definitions and reporting

Adverse event (AE) definition
• An AE is any unfavourable and unintended sign (including abnormal laboratory finding), symptom or disease temporally associated with the use of an investigational product, whether or not related to the investigational product.

Serious adverse event (SAE) definition:
a. Results in death
b. Is life-threatening<br>The term 'life-threatening' in the definition of 'serious' refers to an event in which the participant was at risk of death at the time of the event. It does not refer to an event, which hypothetically might have caused death, if it were more severe.
c. Requires inpatient hospitalization or prolongation of existing hospitalization<br>In general, hospitalization signifies that the participant has been detained (usually involving at least an overnight stay) at the hospital or emergency ward for observation and/or treatment that would not have been appropriate in the physician's office or outpatient setting. Complications that occur during hospitalization are AEs. If a complication prolongs hospitalization or fulfils any other serious criteria, the event is serious. When in doubt as to whether "hospitalization" occurred or was necessary, the AE should be considered serious.<br>Hospitalization for elective treatment of a pre-existing condition that did not worsen from baseline is not considered an AE.
d. Results in persistent disability/incapacity<br>• The term disability means a substantial disruption of a person's ability to conduct normal life functions.<br>• This definition is not intended to include experiences of relatively minor medical significance such as uncomplicated headache, nausea, vomiting, diarrhoea, influenza, and accidental trauma (e.g., sprained ankle) which may interfere with or prevent everyday life functions but do not constitute a substantial disruption.
e. Is a congenital anomaly/birth defect
f. Other situations:<br>• Medical or scientific judgment should be exercised in deciding whether SAE reporting is appropriate in other situations such as important medical events that may not be immediately life-threatening or result in death or hospitalization but may jeopardize the participant or may require medical or surgical intervention to prevent one of the other outcomes listed in the above definition. These events should usually be considered serious.<br>Examples of such events include intensive treatment in an emergency room or at home for allergic bronchospasm or convulsions that do not result in hospitalization, or development of drug dependency or drug abuse.

SAE reporting to FIND
• The SAE Report must be sent to the FIND Head of Programme and Study Manager via e-mail, marked High Priority, with a follow up call to ensure receipt.<br>• Initial notification via telephone does not replace the need for the investigator to complete and sign the SAE Report within the designated reporting time frames.<br>• Contacts for SAE reporting can be found on the front of the protocol.

Appendix 2: Incident definition and reporting

Medical device/IVD incident definition
• A medical device incident is any malfunction or deterioration in the characteristics and/or performance of a device or IVD as well as any inadequacy in the labelling or the instructions for use which, directly or indirectly, might lead to or might have led to the death of a participant/user/other person or to a serious deterioration in his/her state of health.<br>• Not all incidents lead to death or serious deterioration in health. The non-occurrence of such a result might have been due to other fortunate circumstances or to the intervention of health care personnel.

It is sufficient that:

- An incident associated with a device happened.

AND

- The incident was such that, if it occurred again, might lead to death or a serious deterioration in health.

A serious deterioration in state of health can include any of the following:

- Life-threatening illness
- Permanent impairment of body function or permanent damage to body structure
- Condition necessitating medical or surgical intervention to prevent one of the above
- Foetal distress, foetal death, or any congenital abnormality or birth defects

Medical device incident documenting
• Any medical device incident occurring during the study will be documented in the participant's medical records, in accordance with the investigator's normal clinical practice, and on the appropriate form of the CRF.<br>• For medical device incidents fulfilling the definition above, complete the SAE Report Form.<br>• It is very important that the investigator provides his/her assessment of causality (relationship to the medical device provided by FIND) and describes any corrective or remedial actions taken to prevent recurrence of the incident.<br>• A remedial action is any action other than routine maintenance or servicing of a medical device where such action is necessary to prevent recurrence of an incident. This includes any amendment to the device design to prevent recurrence.

Appendix 3: Technical appraisal form

FIND HCV RDT fresh sample study Technical Appraisal Form
28-Feb-2019 v1.0.docx

FIND Because diagnosis matters

Technical Appraisal by Laboratory Technician

**Study name**	Prospective diagnostic accuracy study of Rapid Diagnostic Tests (RDTs) detecting antibodies against hepatitis C virus (HCV) in freshly collected whole blood, plasma and serum
**Protocol number, version, date**	Number: 8162-2/2<br>Version: 1.0<br>Date: 26-Mar-2019
**Technical Appraisal Form version**	1.0
**Author**	Beatrice Vetter; adapted from WHO PQT Report Template for HCV Simple Rapid Assays PHE v6.0 PQDX_137

This form should be completed for each RDT manufacturer by every technician who has performed the RDT

Name of laboratory technician:______________________________ Date:______________

Test name and manufacturer:____

	**Rating***	**1**	**2**	**3**	**4**	**5**
**Kit instructions**	Clarity					
	Presentation					
	Content					
	Safety instructions					
**Kit/reagent packaging and labelling**	Clear					
	Labelling					
	Safety					
**Specimen dispensing and volume**	Specimen type used:					
	Specimen volume (µl):					
	Specimen addition control:					
**Reagent dispensing**	Reagent addition control:					
**Equipment required**	Equipment required:					
	Details of equipment required (optional):					
**Number of steps to test completion**	Number:					
**Endpoint stability**	Minutes:					
**Time from start to completion**	Minutes:					
**Recommended maximum tests per run**	Test maximum:					
**Actual number of tests possible per run**	Test number:					
**Other comments:**						
***Rating key:** 1=poor; 2=needs improvement; 3=satisfactory; 4=good; 5=excellent						

Appendix 4: Summary of changes

The protocol amendment table for the current revision is located directly before the Abbreviations section.

Amendment [amendment number]: [date]

Rationale for the amendment

Section # and title	*Description of change*	*Brief rationale*
