# Supplementary material for: Sensitivity and specificity of rapid hepatitis C antibody assays in freshly collected whole blood, plasma and serum samples: A multicentre prospective study
Source: PLoS One. 2020 Dec 3;15(12):e0243040. doi: 10.1371/journal.pone.0243040 (PMC7714359; doi:10.1371/journal.pone.0243040)
Supplement: Attachment — Submitted filename: HCV fresh sample reviewer response_D1_12Nov.docx [file pone.0243040.s009.docx]

**Reviewer #1**

The authors addressed adequately all reviewers' query. My only last comment regards Supplemental Table 4. It would be useful to add the estimates of the OR and 95% CI and report the exact p-values rather than the cryptic '<0.05'

**Response:** Thank you for this comment, we have now added all p-values down to the third decimal point in Supplemental Table 4. While reviewing the table, we noted that the p-values in the second column for HCV-Ab Rapid compared to the reference RDT were incorrect. We have now corrected this, apologise for the error and note that all statements as made in the manuscript remain correct (lines 245-248).
